# African Swine Fever Virus Uses Macropinocytosis to Enter Host Cells

**DOI:** 10.1371/journal.ppat.1002754

**Published:** 2012-06-14

**Authors:** Elena G. Sánchez, Ana Quintas, Daniel Pérez-Núñez, Marisa Nogal, Susana Barroso, Ángel L. Carrascosa, Yolanda Revilla

**Affiliations:** Centro de Biología Molecular Severo Ochoa, CSIC-UAM, Madrid, Spain; Rosalind Franklin University of Medicine and Science, United States of America

## Abstract

African swine fever (ASF) is caused by a large and highly pathogenic DNA virus, African swine fever virus (ASFV), which provokes severe economic losses and expansion threats. Presently, no specific protection or vaccine against ASF is available, despite the high hazard that the continued occurrence of the disease in sub-Saharan Africa, the recent outbreak in the Caucasus in 2007, and the potential dissemination to neighboring countries, represents. Although virus entry is a remarkable target for the development of protection tools, knowledge of the ASFV entry mechanism is still very limited. Whereas early studies have proposed that the virus enters cells through receptor-mediated endocytosis, the specific mechanism used by ASFV remains uncertain. Here we used the ASFV virulent isolate Ba71, adapted to grow in Vero cells (Ba71V), and the virulent strain E70 to demonstrate that entry and internalization of ASFV includes most of the features of macropinocytosis. By a combination of optical and electron microscopy, we show that the virus causes cytoplasm membrane perturbation, blebbing and ruffles. We have also found that internalization of the virions depends on actin reorganization, activity of Na^+^/H^+^ exchangers, and signaling events typical of the macropinocytic mechanism of endocytosis. The entry of virus into cells appears to directly stimulate dextran uptake, actin polarization and EGFR, PI3K-Akt, Pak1 and Rac1 activation. Inhibition of these key regulators of macropinocytosis, as well as treatment with the drug EIPA, results in a considerable decrease in ASFV entry and infection. In conclusion, this study identifies for the first time the whole pathway for ASFV entry, including the key cellular factors required for the uptake of the virus and the cell signaling involved.

## Author Summary

ASFV is a highly pathogenic zoonotic virus, which can cause severe economic losses and bioterrorism threats. No vaccine against ASFV is available so far. A strong hazard of ASFV dissemination through EU countries from Caucasian areas has recently emerged, thus making urgent to acquire knowledge and tools for protection against this virus. Despite that, our understanding of how ASFV enters host cells is very limited. A thorough understanding of this process would enable to design targeted antiviral therapies and vaccine development. The present study clearly defines key steps of ASFV cellular uptake, as well as the host factors responsible for permitting virus entry into cells. Our results indicate that the primary mechanism of ASFV uptake is a macropinocytosis-like process, that involves cellular membrane perturbation, actin polarization, activity of Na^+^/H^+^ membrane channels, and signaling proceedings typical of the macropinocytic mechanism of endocytosis, such as Rac1-Pak1 pathways, PI3K and tyrosine-kinases activation. These findings help understanding how ASFV infects cells and suggest that disturbance of macropinocytosis may be useful in the impairment of infection and vaccine development.

## Introduction

ASFV is a 200 nm large DNA virus that infects different species of swine, causing acute and often fatal disease [1]–[3]. Infection by ASFV is characterized by the absence of a neutralizing immune response, which has so far hampered the development of a conventional vaccine. A strong hazard of ASFV dissemination through EU countries from Caucasian areas has recently emerged, thus making progress of knowledge and tools for protection against this virus urgent.

Analysis of the complete DNA sequence of the 170-kb genome of the Ba71V isolate, adapted to grow in Vero cells, has revealed the existence of 151 genes, a number of enzymes with functions related to DNA replication, gene transcription and protein modifications, as well as several genes able to modulate virus-host interaction [4]–[12].

ASFV replicates within the host cell cytosol, although a nuclear step has been reported [13], [14]. Discrete cytoplasmic areas are reorganized into viral replication sites, known as factories, during the productive virus cycle. Regarding this, we have recently described ASFV replication as fully dependent on the cellular translational machinery since it is used by the virus to synthesize viral proteins. Thus, during infection, factors belonging to the eukaryotic translational initiation complex eIF4F are phosphorylated, and then redistributed to the periphery of the ASFV factory. Furthermore, ASFV late mRNAs, ribosomes and mitochondrial network were also located in these areas [15]. Such phosphorylation events and redistribution movements suggest, first, a reorganization of the actin skeleton induced by ASFV, and second, virus-dependent kinases activation mechanisms. Several other critical steps of the infection, probably including virus entry and trafficking, might be also regulated by phosphorylation of key molecules targeted by the virus.

As the first step of replication, entry into the host cell is a prominent target for impairing ASFV infection and for potential vaccine development. Endocytosis is a major pathway of pathogen uptake into eukaryotic cells [16]. Clathrin-mediated endocytosis is one of the best studied receptor-dependent pathways, characterized by the formation of clathrin coated pits of 85–110 nm in diameter that bud into the cytoplasm to form clathrin-coated vesicles. Relatively low size viruses, as Vesicular stomatitis virus, Influenza virus, and Semliki forest virus all enter their host cells using this mechanism [17]–[19]. On the other hand, the caveolae-mediated pathway is dependent on small vesicles termed caveolae (50–80 nm) enriched in caveolin, cholesterol, and sphingolipid. It has been implicated in the entry of other small viruses such as Simian virus 40 [20].

Macropinocytosis is another important type of endocytic route used by several viruses to enter host cells. It is defined as an actin-dependent endocytic process associated with a vigorous plasma membrane activity in the form of ruffles or blebs induced by activation of kinases and Rho GTPases. This pathway involves receptor- independent internalization of fluid or solutes into large uncoated vesicles sized between 0.5–10 µm called macropinosomes [21], [22]. In recent years, it has been reported that macropinocytosis is responsible for virus entry of Vaccinia virus (VV) [23], [24], Coxsackievirus [25], Adenovirus-3 [26], Herpes simplex virus [27]–[29], and is required for other viruses to promote viral internalization after entry by some different endocytic mechanism [30]–[32].

Regarding ASFV entry, preliminary studies were reported many years ago by our lab describing this process as temperature, energy, cholesterol and low pH-dependent, and also showing that ASFV strain Ba71V enters Vero cells by receptor-mediated endocytosis [33]–[37]. However, the cellular molecules involved and the precise mechanisms for ASFV entry remain largely unknown.

A recent paper [38] reported that ASFV uses dynamin and clathrin-dependent endocytosis to infect cells. However, it is noteworthy that this work employed the expression of ASFV early proteins as readout of virus entry, which is not equivalent to virus uptake, since several post-entry events could be involved in virus early protein expression. Hence, explanation of several controversial points, such as the larger size of ASFV (200 nm) compared to the smaller size (50–80 nm) of clathrin coated pits, or the existence of several other possible roles for dynamin in addition to virus entry [39], are not discussed in that work.

In the present work we have characterized the mechanisms of entry of ASFV-Ba71V and ASFV-E70 strains either in Vero or swine macrophages, as representative models for ASFV infection. By means of a combination of pharmacological inhibitors, specific dominant-negatives and confocal and electron microscopy, we show that ASFV is taken up predominantly by macropinocytosis. Therefore, we provide evidence, for the first time, that the ASFV entry requires sodium/proton exchanger (Na^+^/H^+^), activation of EGFR and PI3K, phosphorylation of Pak1 kinases together with activation of Rho-GTPase Rac1 and relies on actin-dependent blebbing/ruffling formation, all events fully linked with macropinocytosis activation.

## Materials and Methods

### Cell culture, viruses and infections

Vero (African green monkey kidney) cells were obtained from the American Type Culture Collection (ATCC) and grown in Dulbecco's Modified Eagle's Medium (DMEM) supplemented with 5% fetal bovine serum (Invitrogen Life Technologies). IPAM cells (porcine macrophage-derived cell lines) were kindly provided by Dr. Parkhouse (Fundaçao Calouste Gulbenkian - Instituto Gulbenkian de Ciência, Oeiras, Portugal) and grown in RPMI 1640 medium supplemented with 10% fetal bovine serum. Cells were grown at 37°C under a 7% CO_2_ atmosphere saturated with water vapour in a culture medium supplemented with 2 mM L-glutamine, 100 U/ml gentamicin and nonessential amino acids. The Vero-adapted ASFV strain Ba71V and isolate E70 were propagated and titrated by plaque assay on Vero cells, as described previously [40], [41]. In brief, subconfluent Vero cells were cultivated in roller bottles and infected with ASFV at a multiplicity of infection (MOI) of 0.5 in DMEM 2% fetal bovine serum. After 72 h post infection the cells were recovered and centrifuged at 3000 rpm for 15 min and the cellular pellet was discarded. The supernatant containing viruses was clarified at 14000 rpm for 6 h at 4°C and the purified infectious virus was resuspended in medium and stored at −80°C. Vero cells were infected with Ba71V isolate and IPAM cells with E70 or Ba71V as indicated. The MOI used ranged from 1 to 3000 pfu/cell, as explained.

Viral adsorption to cells was performed at 4°C (synchronic infection) or at 37°C (asynchronic infection) during 90 min (or 60 min when indicated), followed by one wash with cold PBS, and a shift to 37°C to allow the infection until indicated times.

### Pharmacological inhibitors and antibodies

Pharmacological inhibitors were prepared either in water or DMSO following the manufacturer's recommendation and used at the indicated concentration. 5- ethylisopropyl amiloride (EIPA), Cytochalasin D (Cyto D), Genistein, IPA-3, Chlorpromazine (CPZ), Dynasore (Dyn) and Nocodazole were purchased from Sigma. ±Blebbistatin, EGFR inhibitor (324674) and Rac1 inhibitor (Rac1 Inh, NSC23766) was purchased from Calbiochem, and LY294002 (LY) from Echelon.

Specific antibodies against Akt, phospho-Akt (Thr308), phospho-Akt (Ser473) and PI3K p85 were purchased from Cell Signaling Technology; anti-Pak1, anti- phospho-Pak1 (Thr423), anti-Rock1 and anti-β-actin from Santa Cruz Biotechnology, Inc. Rac1 was detected with a monoclonal antibody from Millipore, kindly provided by Dr. C. Murga (CBMSO, Madrid, Spain). Monoclonal anti-p72 (17LD3) [42] was a kind gift from Ingenasa and polyclonal antibodies risen against p72, p32 and most of the ASFV structural proteins (anti-ASFV) were generated in our laboratory. Alexa Fluor 594-WGA, TRITC- phalloidin, Alexa Fluor 488-phalloidin, Topro3, anti-mouse Alexa Fluor-488, anti-goat Alexa Fluor-555 and anti-mouse Alexa Fluor-555 were purchased from Invitrogen, and anti-rabbit, anti-mouse and anti-goat immunoglobulin G coupled to peroxidase from Amersham Biosciences.

### Plasmids construct

GFP-tagged versions of wild type forms of actin (pEGFP-actin) and Rac1 (pEGFP-Rac1) were kindly provided by Dr. J. Mercer (ETH Zurich, Institute of Biochemistry, Zurich, Switzerland) and Rac1 mutant form (pGFP-Rac1-N17) was a generous gift from Dr. R. Madrid (CBMSO, Madrid, Spain). GFP-tagged versions of WT, AID, and T423E of Pak1 constructs were a gentle gift from Dr. J. Chernoff (Fox Chase Cancer Center, Philadelphia, PA, USA) and pEGFP-C2 was purchased from Invitrogen.

### ASFV uptake and infection assays

To analyze ASFV uptake, Vero cells were pretreated with the pharmacological inhibitors listed above at 37°C for 60 min in serum free medium. Ba71V synchronic infection was carried out at a MOI of 10 pfu/cell in the presence of the drugs. After binding, cells were washed once with cold PBS, followed by the addition of containing drug medium, and infection was allowed to proceed for 60 min at 37°C. After infection, cells were fixed and prepared either for Fluorescence Activating Cell Sorting (FACS) or Confocal Laser Scanning Microscopy (CLSM) analysis.

The specific effect of the drugs on virus entry and post entry steps was analyzed by incubation of the cells either 60 min before virus addition or 60 min after virus addition, and viral infection was allowed in the presence of the drugs at 37°C, in each case. Ba71V or E70 asynchronic infection was carried out for 16 or 48 h at a MOI of 1 pfu/cell or at a MOI of 5 pfu/cell to analyze viral proteins by Western blot or number of infected cells by CLSM, respectively.

To analyze Akt phosphorylation upon ASFV infection, Vero cells were infected at a MOI of 10 pfu/cell and viral adsorption was allowed for 60 min at 37°C. Actin distribution analysis was performed at different times post infection since virus addition at 37°C at MOI 50. Rac1 distribution and Pak1 phosphorylation was measured after synchronic infection at a MOI of 10 pfu/cell. At the indicated times, cells were prepared for Western blot or CLSM analysis.

### Viral production assays

Vero cells were pretreated with DMSO or pharmacological inhibitors for 60 min at 37°C. The asynchronic infection was carried out at a MOI of 1 pfu/cell for 48 h in the presence of the inhibitors and the supernatant was recovered. The number of productive viral particles was titrated by plaque assays on Vero cells as described in [41].

### Field Emission Scanning Electron Microscopy (FESEM)

Cells were grown on glass coverslips, serum starved for 24 h, infected synchronously (MOI 50) and at the indicated times post infection, fixed in 2.5% glutaraldehyde and 4% paraformaldehyde in 0.1 M phosphate buffer (pH 7.4) for 3 h at 4°C. They were washed three times in phosphate buffer, postfixed in 2% OsO_4_/water at RT for 60 min, washed in water, dehydrated in acetone, critical point dried for 2 h and coated with graphite-gold in a sputter coater. The samples were analyzed with a JSM-6335-F (JEOL) Field Emission SEM (Electron Microscopy National Center, UCM; Madrid, Spain).

### Transmission Electron Microscopy (TEM)

Vero cells were serum starved 24 h and virus binding was allowed for 90 min at 4°C with Ba71V (MOI 3000). Cells were fixed with 2% glutaraldehyde and 4% paraformaldehyde in 0.1 M phosphate buffer (pH 7.4) for 3 h at 4°C. Sections of infected cells were prepared as described [43] and analyzed in a JEOL 100B electron microscope.

### Phase Contrast Microscopy and Nomarski

In order to study real-time live imaging of ruffles formation induced by ASFV infection, Vero cells were serum starved for 24 h and virus binding was allowed for 90 min at 4°C at MOI 100. After binding, cells were washed with cold PBS and images were collected for 30 min with an Orca R2 digital camera (Hamamatsu) on a wide-field microscope (LeicaDMI6000B, Leica Microsystems) with controlled environmental chamber (temperature 37°C and 5% CO_2_ humidified atmosphere). Images were captured with LAS AF version 2.6.0 software (Leica Microsystems) at a resolution of 1344×1024 pixels using a 20×, 0.40 NA objective with a 1.6× magnification-changer, and analyzed with Image J software.

To analyze blebs formation, IPAM cells were infected synchronously (MOI 50) and at different times post infection, fixed with paraformaldehyde 4% for 20 min. Images were taken with a ccd monochrome camera (Hamamatsu) on a invert microscope (Axiovert200, Zeiss) using a 63× objective and analyzed with Image J program.

### Fluorescence Activated Cell Sorting (FACS)

Mock-infected or infected cells in the presence of pharmacological inhibitors were detached with trypsin-EDTA after 60 min post infection (mpi), fixed with 2% paraformaldehyde for 30 min at 4°C and then permeabilized with PBS-Staining buffer (PBS 1×, 0.01% sodium azide, 0.5% BSA) 0.2% saponin for 15 min at RT. Detection of infected cells was performed by incubation with an anti-p72 monoclonal antibody (17LD3) (diluted 1∶100 in PBS-Staining buffer 0.2% saponin) for 20 min at 4°C, followed by incubation with an anti-mouse Alexa Fluor-488 (diluted 1∶500 in PBS-Staining buffer, 0.2% saponin) in the same conditions. Finally, 2×10^4^ cells were analyzed in a FACSCalibur flow cytometer (BD Science) to determine the percentage of infected cells. All FACS analyses were performed at least in triplicate and displayed as the average percentage of infected cells relative to control infection in the absence of a pharmacological inhibitor. Error bars represent the standard deviation between experiments.

### Confocal Laser Scanning Microscopy (CLSM)

Cells were grown on glass coverslips and, at indicated times post infection, were fixed with 4% paraformaldehyde for 20 min and permeabilized with PBS-0.2% Triton X-100 for 30 min at RT. Viral particles or infected cells were stained with an anti-p72 monoclonal antibody (17LD3) (diluted 1∶250 in PBS-5% BSA) for 60 min at RT, followed by incubation with an anti-mouse Alexa Fluor-488 or an anti-mouse Alexa Fluor-555 for the same time. Alexa Fluor-488 phalloidin (dilution 1∶100) or TRICT- phalloidin and Topro3 (dilution 1∶500) were used to stain actin filaments and nuclei of cell, respectively. Goat anti-Rock1 was used at a dilution 1∶50.

To analyze the virus binding to the cellular membrane, the viral adsorption was allowed for 90 min at 4°C (MOI 10) and after 60 min from virus addition cells were incubated with Alexa Fluor 594-WGA for 30 min. Cells were washed twice with cold PBS-0.1% BSA Buffer and incubated with anti-p72 monoclonal antibody (17LD3) and Alexa Fluor-488 for 60 min at 4°C. Finally, cells were fixed with 4% paraformaldehyde at RT for 20 min.

Samples were analyzed by CLSM (Zeiss LSM510) with a 63× oil immersion objective. To investigate ASFV uptake as well as actin, Rock1 and Rac1 distribution, Z-slices per image were collected and displayed as maximum z-projection of vertical slices (*x–z* plane) and/or maximum z-projection of horizontal slices (*x–y* plane). For presentation of images in the manuscript, LSM images were imported into Image J software for brightness and contrast enhancements. In all instances one image is representative of three independent experiments.

ASFV uptake in the presence of inhibitors was analyzed automatically by a Macro algorithm from Image J program (developed by CBMSO Confocal Microscopy Service, Spain) in which Intermode threshold was used to count the number of virus inside the cells.

### Fluid phase uptake assays

Vero cells were serum starved for 24 h and pretreated with DMSO or EIPA. After 60 min at 37°C the cells were synchronously infected (MOI 10) or treated with PMA (200 nM) at 37°C for 30 min. Fifteen min prior to harvesting or fixation, cells were incubated with 0.5 mg/ml 10 KDa 647-dextran or 3 KDa Texas Red-dextran (Invitrogen) at 37°C. Dextran uptake was stopped by placing the cells on ice and washing three times with cold PBS and once with low pH buffer (0.1 M sodium acetate, 0.05 M NaCl, pH 5.5) for 10 min. Then, the cells were prepared for FACS or CLSM analysis. In FACS experiments dextran uptake was displayed as fluorescence mean of three independent experiments. Error bars represent the standard deviation between experiments. Cells without wash acid buffer were added as an experiment control.

### Western blot analysis

At indicated times post infection, cells were washed with PBS and lysed in RIPA modified buffer (50 mM Tris-HCl pH 7.5, 1% NP40, 0.25% Na-deoxycolate, 150 mM NaCl, 1 mM EDTA) supplemented with protease and phosphatase inhibitor cocktail tablets (Roche). The protein concentration was determined by a Pierce BCA Protein Assay kit based on the bicinchoninic acid spectrophotometric method (Thermo Scientific). Cell lysates (15–50 µg of protein) were fractionated by SDS-PAGE and electrophoretically transferred to an Immobilon extra membrane (Amersham) and the separated proteins reacted with specific primary antibodies. The antibodies used were the following: polyclonal anti-p72 (dilution 1∶2000), anti-p32 (dilution 1∶1000), anti-ASFV (dilution 1∶3000); anti-Akt, anti-phospho Akt (Thr308), anti-phospho Akt (Ser473), anti-Pak1 and anti-phospho Pak1 (Thr423) (dilution 1∶500); anti-β-actin and anti-Rac1 (dilution 1∶1000). Membranes were exposed to horseradish peroxidase-conjugated secondary antibodies (dilution 1∶5000) followed by chemiluminescence (ECL, Amersham Biosciences) detection by autoradiography. In all instances the figures are representative of three independent experiments.

### PI3K activation assay

Vero cells were serum starved for 24 hours and treated with DMSO or LY294002 for 60 min at 37°C in serum free medium. Asynchronic infection (viral adsorption for 60 min) was carried out at a MOI of 10 pfu/cell in the presence of the drug at 37°C until indicated times. PI3K subunit p85 was immunoprecipitated from lysed cells and PI3-kinase activity was measured as PI(3,4,5)P_3_ production by ELISA activation kit, following the manufacturer's recommendations (Kit#1001s Echelon).

### Rac1 activation assays

Vero cells were serum starved for 24 hours before synchronic infection at a MOI of 10 pfu/cell. The cells were washed once with cold PBS, shifted to 37°C and harvested at the indicated times post infection. Rac1 activation was measured with a G-LISA activation kit (Kit #BK128 Cytoskeleton, Inc.) and by immunoblotting after a Pak1-PBD-Agarose Beads (Upstate) pull down step as described following the manufacturer's recommendations. Bound Rac1-GTP was detected by incubation with an anti-Rac1 specific antibody followed by a secondary antibody conjugated to HRP and a detection reagent. The signal was read by measuring absorbance at 490 nm using a microplate reader and by autoradiography.

### Acid-mediated endocytosis by-pass assay

To check if the EIPA inhibitor was specifically blocking virus entry and not a down-stream process such as early gene expression, we induced the fusion of the viral membrane with the plasma membrane (PM) by lowering the pH of the medium [23]. The cells were pretreated with EIPA for 60 min at 37°C in serum free medium. Viral adsorption was allowed at MOI 1 for 90 min at 37°C in neutral (7.4) or acid (5.0) pH. Cells were washed once with cold PBS and infection was allowed to proceed for 16 h at 37°C in the presence of the inhibitor in neutral pH. Samples were prepared for Western blot analysis.

### Transfection assays

Vero cells were transfected with 1 µg of specific expression plasmids per 10^6^ cells using the *LipofectAMINE Plus Reagent* (Invitrogen) according to the manufacturer's instructions and mixing in *Opti-MEM* (Invitrogen) in a 6-well plate. Cells were incubated at 37°C for 4 h in serum free medium, washed and incubated at 37°C. After 16 or 24 h post transfection the cells were infected at indicated MOI and either lysated and analyzed by Western blot, or fixed and prepared for CLSM analysis.

### P72 protein detection in purified viruses

In order to analyze the localization of p72 in the viral particle, we carried out an experimental procedure as described in [44]. In brief, purified virus was treated with different buffers (Buffer 1 and 2) for 30 min at RT and the separate samples were centrifugated over sucrose (20% in PBS) cushion in a Beckman Airfuge at 24 p.s.i. for 15 min. The supernatant (SP) and pellet (P) were analyzed by Western blot by incubation with anti-p72 monoclonal antibody (17LD3). Buffer 1: 10 mM Tris pH 8, 0.65 M NaCl, 0.5% octil β- D- Glucopyranoside (Sigma); Buffer 2: 10 mM Tris pH 8, 0.65 M NaCl, 0.2% octil β- D- Glucopyranoside (Sigma) and 0.1% Dithiothreitol (DTT).

### Toxicity analysis by Trypan Blue

To check cell viability after treatment with inhibitors cells were dyed with Trypan Blue and dead cells were counted in hemocytometer as blue cells.

### Densitometry analysis

After Western Blot analysis, bands developed by ECL chemiluminescence were digitalized by scanning and quantified with Fujifilm Multi Gauge V3.0 software. Data were normalized after subtracting background values and calculated as factors by their ratio against the highest or lowest positive value obtained. All quantifications represent the mean of three independent experiments.

### Accession numbers

**ASFV proteins in Swiss Prot database**: **p72**: MCP_ASFB7; **p32**: P30_ASFB7; **p17**: P17_ASFB7; **p12**: P12_ASFB7.

**Cellular proteins in ENSEMBL database**: **Pak1**: ENSMMUG00000001387; **Rac1**: ENSFM00250000002337; **β-actin**: ENSMMUG00000012054; **Akt**: ENSMMUG00000001041; **Rock1**: ENSFM00540000717933.

## Results

### ASFV induces membrane ruffles and blebs to enter host cells

Macropinocytosis mainly differs from other endocytic processes in the requirement of extensive actin cytoskeleton restructuring and formation of blebs or ruffling in the cellular surface, through which the specific cargo enters the cell [22]. These rearrangements are coupled to an external-induced formation of plasma membrane extensions. Several viruses have been described to use macropinocytosis for entry, including Vaccinia virus [23], [45], [46], Ebola virus [47] and Kaposi's sarcoma-associated herpesvirus [29], [48].

**Figure 1:**
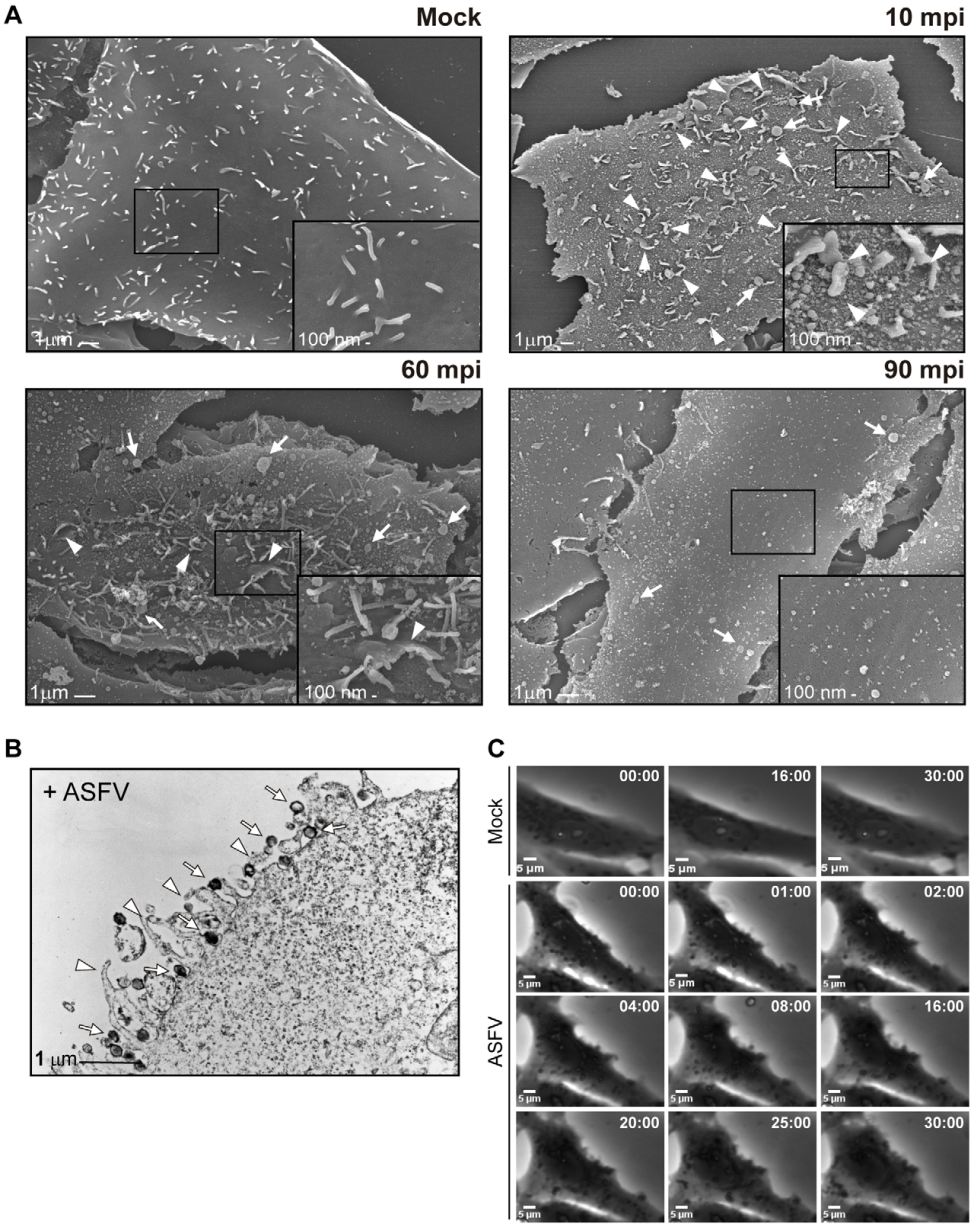
Ruffles induction upon ASFV entry in Vero cells. **A**) Field Emission SEM of mock-infected and infected cells. Cells were serum starved for 24 h and synchronously infected for 10, 60 and 90 min (MOI 50). A magnification of the cell surface detail (boxes) is shown in the lower right panels. Arrowheads indicate ruffles and arrows indicate bubble-like membrane perturbations. **B**) TEM of purified viral particle (arrows) localized into ruffles (arrowheads) in the cells after binding for 90 min at 4°C (MOI 3000). **C**) Ruffle induction upon ASFV binding to Vero cells. After being serum starved for 24 h, virus binding was allowed for 90 min at 4°C (MOI 100), and infected cells were recorded during 30 min after warming at 37°C with a 20× objective. Time stamps indicate min: sec.

Receptor-mediated endocytosis has been postulated in classic studies as the most likely mechanism for ASFV entry into Vero cells [33]–[35]. Yet the specific characteristics to further depict the viral entry procedure have not been elucidated. To analyze the possible perturbation of the cellular membrane induced by ASFV, the virus strain Ba71V was used to synchronously infect Vero cells at MOI 50. To achieve this, we have analyzed by Field Emission SEM analysis (FESEM) the induction of ruffling and bubbles-like perturbations at 10, 60 and 90 min after ASFV uptake. The results are shown in Figure 1A, where a maximum level of membrane perturbation similar to ruffles appears in ASFV-infected Vero cells between 10 and 60 mpi, decreasing after 90 mpi, indicating that ASFV-induced macropinocytosis is a transient event.

On the other hand, Figure 1B shows that ASF virions internalize in Vero cells adjacent to retracting ruffles, thus indicating that the macropinocytic uptake of viral particles seems to occur as part of the macropinocytic process.

Finally, we have analyzed *in vivo* in real-time the membrane protrusions observed during Ba71V infection in Vero cells. Figure 1C shows the sequence of images during the first minutes of the infection (Video S2), illustrating the ASFV-induced ruffling, and in concordance with the data shown in Figure 1A. For comparison to Mock-infected Vero cells, see Video S1.

**Figure 2:**
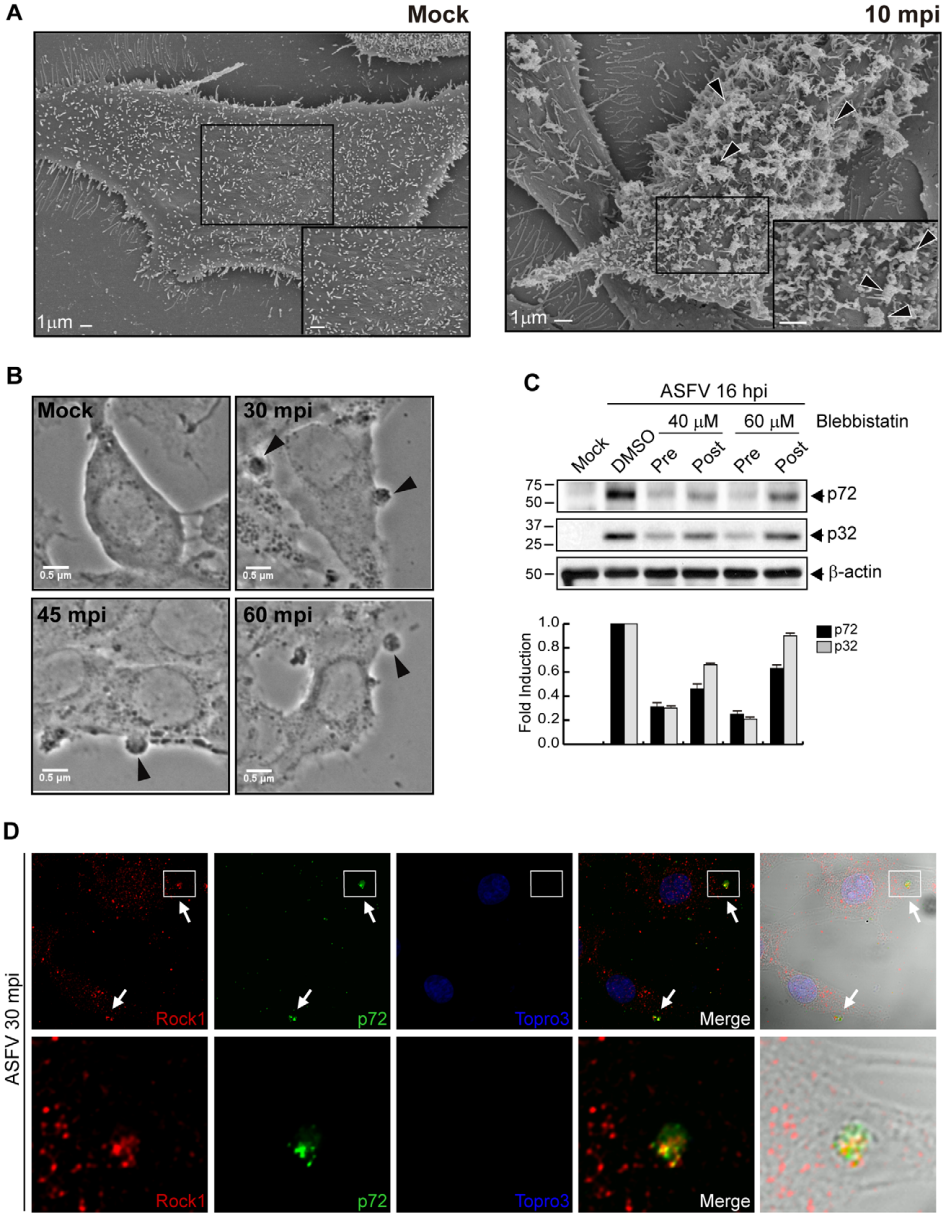
Blebs induction upon ASFV entry in IPAM cells. **A**) Field Emission SEM of mock-infected and infected cells. Cells were synchronously infected for 10 min (MOI 50) with E70 after serum starved for 24 h. Membrane perturbations are indicated by arrowheads. A magnification of the cell surface detail (boxes) is shown in the lower right panels. Bars: 1 µM. **B**) After synchronic infection at different times (E70, MOI 50), the cells were fixed and blebs formation (arrowheads) was analyzed by Phase Contrast Microscopy using a 63× objective. **C**) Blebbistatin treatment inhibits ASFV entry. Cells were treated with DMSO or Blebbistatin 60 min before the infection (Pre) or treated 60 min after virus addition (Post) and maintained during the infection, at indicated concentrations. After 16 hpi (Ba71V, MOI 1) equivalent amounts of protein were analyzed by immunoblotting with an anti-ASFV antibody. β-actin was detected as a load control. Fold induction was determined by densitometry (mean ±S.D) as shown in the graphic below. **D**) Rock1 colocalizes with ASFV in blebs (arrows). Cells were infected (Ba71V, MOI 50) and fixed at 30 min after infection. Cells were incubated with, anti-Rock1 (red), anti-p72 (green) and Topro3 (blue) to stain blebs, virus and nuclei, respectively. Images were taken by CLSM and represented as a maximum z-projection of x–y plane and Normasky. Magnifications of the bleb containing Rock1 and viruses (boxes) are shown in the corresponding bottom panels. S.D., standard deviations.

To assess whether the ASFV entry also induces membrane perturbation in swine macrophages, the natural target cell of ASFV infection *in vivo*, the virulent strain E70 was used to synchronously infect IPAM cells at MOI 50. As early as 10 mpi, strong membrane protrusions were observed by FESEM analysis (Figure 2A). To better characterize these membrane rearrangements, IPAM cells were synchronously infected with E70 strain at MOI 50, during 30, 45 and 60 mpi. Next, IPAM cells were fixed and analyzed by optic microscopy. Figure 2B shows images compatible with blebs induced by ASFV infection in swine macrophages from 30 mpi. To prove this point, we achieved an additional experiment showing the inhibition of virus entry with different doses of blebbistatin, an inhibitor of blebbing and macropinocytosis [23], [29], [49]–[51]. Western blot analyses have shown that blebbistatin impairs the entry of the virus in IPAM cells, as the drug inhibits the expression of ASFV proteins when preincubated before virus addition. Hence, when blebbistatin was incubated 60 min after virus addition, a much lower inhibition of viral proteins was observed, thus indicating the role of blebbistatin on early steps of virus entry. Results are presented in Figure 2C.

Last, by using a specific anti-Rock1 antibody as a marker of blebs [52], we have shown that Rock1 colocalizes with virus particles on blebs in IPAM cells from 30 min after ASFV uptake (Figure 2D), revealing the close relation between bleb and viral particle.

Taken together, these data strongly indicate that ASFV induces a vigorous plasma membrane activity during the first steps of the infection, both in Vero and IPAM cells, well-matching with macropinocytosis-mediated entry.

### ASFV entry is dependent on Na^+^/H^+^ membrane exchangers and stimulates uptake of fluid phase markers

With the membrane perturbation pattern shown above, it was likely that ASFV was using macropinocytosis to enter cells. Macropinocytosis is dependent on the Na^+^/H^+^ exchanger [21], and thus amiloride and its analogue 5-(N-ethhyl-n-isopropil)-amiloride (EIPA) are frequently used as the main diagnostic test to identify macropinocytosis because this drug has been shown to be specific to this endocytic pathway without affecting others [53]–[55]. Consequently, to further assess the involvement of macropinocytosis in ASFV entry, the effect of EIPA was investigated. When tested on Vero cells, EIPA had no significant cytotoxic effect as assessed by cell monolayer integrity and trypan blue cell viability assessment (Table S1).

**Figure 3:**
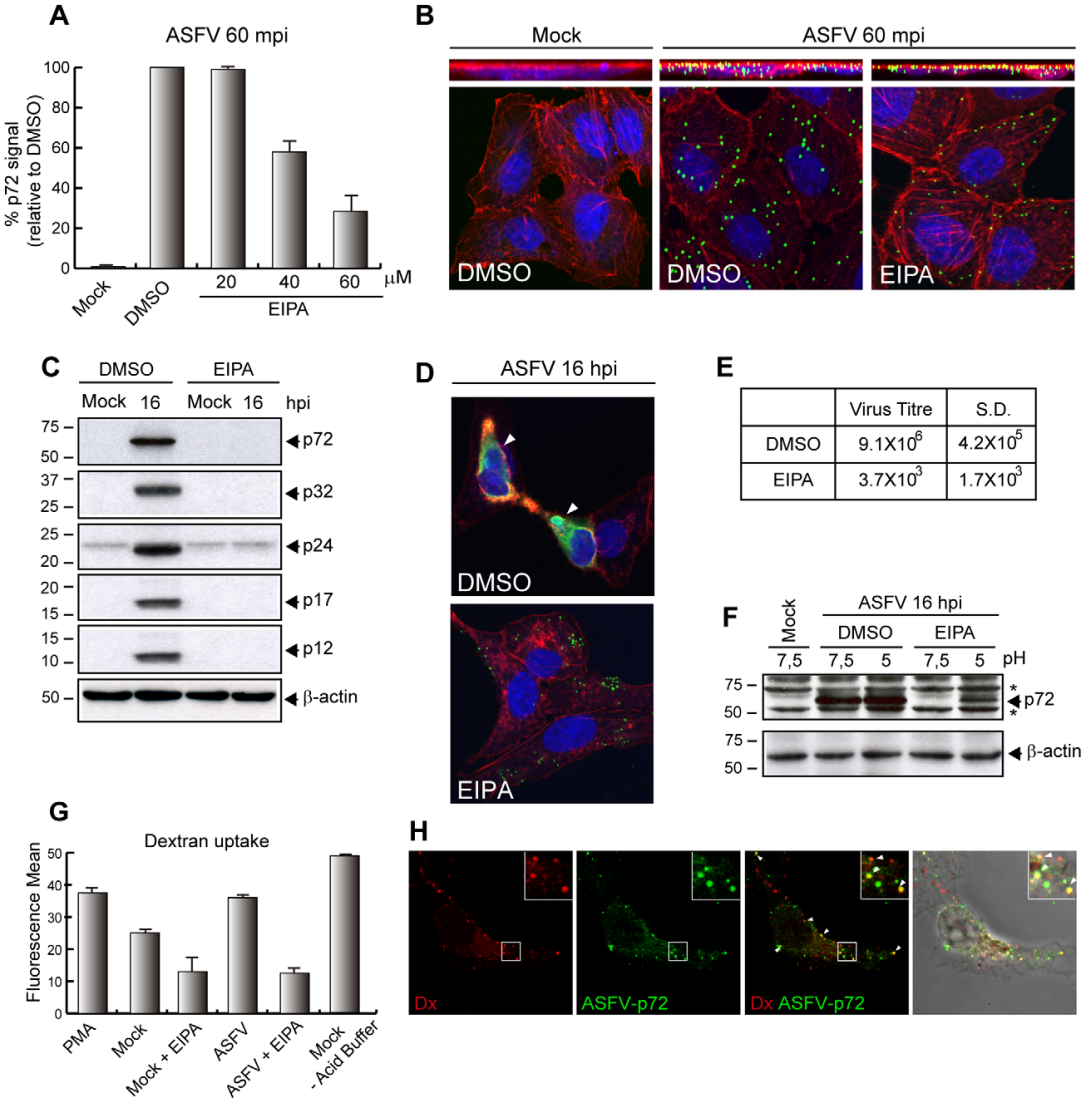
EIPA treatment inhibits ASFV entry and infection in Vero cells. **A–B**) EIPA inhibits ASFV uptake. Cells were pretreated (DMSO or EIPA) and infected (MOI 10) for 60 min. **A**) Infected cells were analyzed by FACS. Graphic shows percentage of virus entry relative to DMSO control, measured as p72 signal. (n = 7, performed in duplicate; mean±S.D.) **B**) Cells were incubated with Topro3, TRITC-phalloidin and anti-p72 to stain nuclei (blue), actin filaments (red) and viral particles (green), respectively. Images were taken by CLSM and represented as a maximum z- projection of x–y plane (bottom panels) and x–z plane (upper panels). **C–E**) The infection is inhibited by EIPA. **C**) Pretreated cells (20 µM EIPA) were infected (MOI 1) for 16 h and analyzed by immunoblotting with an anti-p72 and an anti-ASFV polyclonal antibodies. **D**) Pretreated cells (60 µM EIPA) were infected (MOI 5) and stained with Topro3, TRITC-phalloidin and anti-p72. Images were taken by CLSM (mid z-section). Arrowheads: viral factories. **E**) Supernatants from pretreated (20 µM EIPA) and infected cells (MOI 1) were recovered and lytic viruses were titrated (n = 3, mean ±S.D). **F**) Bypass of EIPA of ASFV infectivity. Acid mediated bypass was performed and samples of pretreated (20 µM EIPA) and infected cells (MOI 1) for 16 h were analyzed by immunoblotting with an anti-ASFV antibody. **G–H**) ASFV colocalizes with dextran and induces its uptake. **G**) Cells were pretreated (60 µM EIPA) and infected (MOI 10) or stimulated with PMA for 30 min, pulsed with 647-dextran for 15 min and analyzed by FACS (n = 3; mean ±S.D.). **H**) After 30 mpi cells were pulsed with Texas-red-dextran for 15 min and incubated with anti-p72 antibody. Images were taken by CLSM (mid z-section) and Nomarsky. Arrowheads: dextran-virus colocalization. β-actin: load control. S.D., standard deviations. ^*^ Unspecific cellular protein detected by the antibody.

It has been previously described that after 60 mpi more than 90% of the ASF viral particles are located in the cell [34]. Furthermore, the viral uncoating does not completely occur before 2 hours post infection (hpi) [34]. According to these data, we measured viral uptake by using the specific antibody 17LD3 against p72, the major protein of ASFV capsid [42], [56] (see Materials and Methods and Figure S1A, B and C). Interestingly, amounts of EIPA from 40 µM to 60 µM caused a significant reduction (60%) in the uptake of ASFV infective particles after 60 mpi (Figure 3A), suggesting that ASFV entry depends on Na^+^/H^+^ exchanger activity/function.

To further visualize the effect of EIPA on virus uptake, Ba71V strain was added to Vero cells, previously treated with DMSO or 60 µM EIPA. Sixty min after infection, the cells were incubated with anti-p72 antibody 17LD3 to stain the virus. A confocal microscopy analysis revealed that there was a noticeable drop in virus particles incorporated into the cells incubated with EIPA, as compared to those incorporated into DMSO-incubated cells (Figure 3B, bottom panels). Images were taken as a maximum z-projection (x–y plane). For clarification, individual channels are shown in Figure S2A. Moreover, we also analyzed images of a maximum z-projection of vertical slices to determine whether viral particles could be imbibed into the membrane in the presence of the inhibitor. As shown in the Figure 3B upper panels, a different distribution of viral particles in the cells infected in the presence of EIPA, compared to that found in cells infected in the absence of the drug, was observed. This last data strongly suggests that in EIPA-treated cells the virus can bind to the membrane but is not able to internalize. This could be the explanation for the percentage of cells that were positive for 17LD3 antibody detected in Figure 3A. The total number of virus obtained in the confocal images was automatically quantified using a macro algorithm in the Image J program (Figure S3).

In regard to this, it is also remarkable that, although a small amount of viral particles can still be detected inside the cells in the presence of EIPA, neither early, p32, nor late ASFV proteins, p17, p24, p12 and p72 [57]–[60] could be detected by Western blot in the presence of the drug (Figure 3C). Hence, it is likely that EIPA is mainly affecting virus uptake since when drug is added 60 min after virus uptake, it does not affect the viral protein synthesis (Figure S4A). As expected, no viral factories detected by using anti-p72 antibody (green) and Topro3 (blue) for viral and cellular DNA, were found after EIPA treatment by confocal microscopy (Figure 3D). Separate channels are shown in Figure S2B and a morphological detail of an ASFV factory is shown in Figure S1C. Consequently, viral production was also strongly inhibited by the drug (Figure 3E). Finally, and to fully ascertain if EIPA was specifically blocking ASFV entry and not a downstream step, we performed the infection by using the acid-mediated fusion of plasma membrane. Briefly, in the presence of acid pH, endocytosis is subverted and virions fused with the plasma membrane and then directly carried into the cytosol. When an inhibitor blocks virus endocytosis, inhibition of viral protein synthesis in the presence of drug can be bypassed through fusion. If membrane fusion could not rescue viral gene expression, the blocking would most probably occur at a post-entry step [23]. By using this method, we find that when the viral adsorption is performed in the presence of EIPA in acidic pH, p72 viral synthesis is clearly recovered in relation to the infection developed at neutral pH (Figure 3F).

Next, we investigated the dextran uptake during ASFV infection, since it has been described that macropinocytosis activation induces a transient increase of this fluid phase marker [61], [62]. To achieve this, Vero cells were treated with EIPA for 60 min and then infected synchronously with Ba71V for 30 min, or stimulated with PMA as a positive control. Fifteen minutes before stopping the infection, cells were pulsed with dextran and prepared for FACS analysis. As indicated in Figure 3G, ASFV infection induces dextran uptake during the virus entry and this action is inhibited by EIPA. Moreover, to reinforce the hypothesis that ASFV entry occurs mainly by macropinocytosis, we developed an experiment to assess the colocalization between the virus particles and the macropinocytosis marker dextran. These results are included in Figure 3H.

All together, these data strongly indicate that ASFV induces activation of macropinocytosis to enter cells.

### Chemical disruption of actin cytoskeleton inhibits ASFV entry

Macropinocytosis is a very specific actin-dependent endocytic process since it depends on acting rearrangements to induce membrane ruffling formation, and inhibitors of actin microfilaments, such as Cytochalasin D (Cyto D) [63], [64], Latrunculin A [65] and Jasplakinolide [66], are commonly used to inhibit this process.

**Figure 4:**
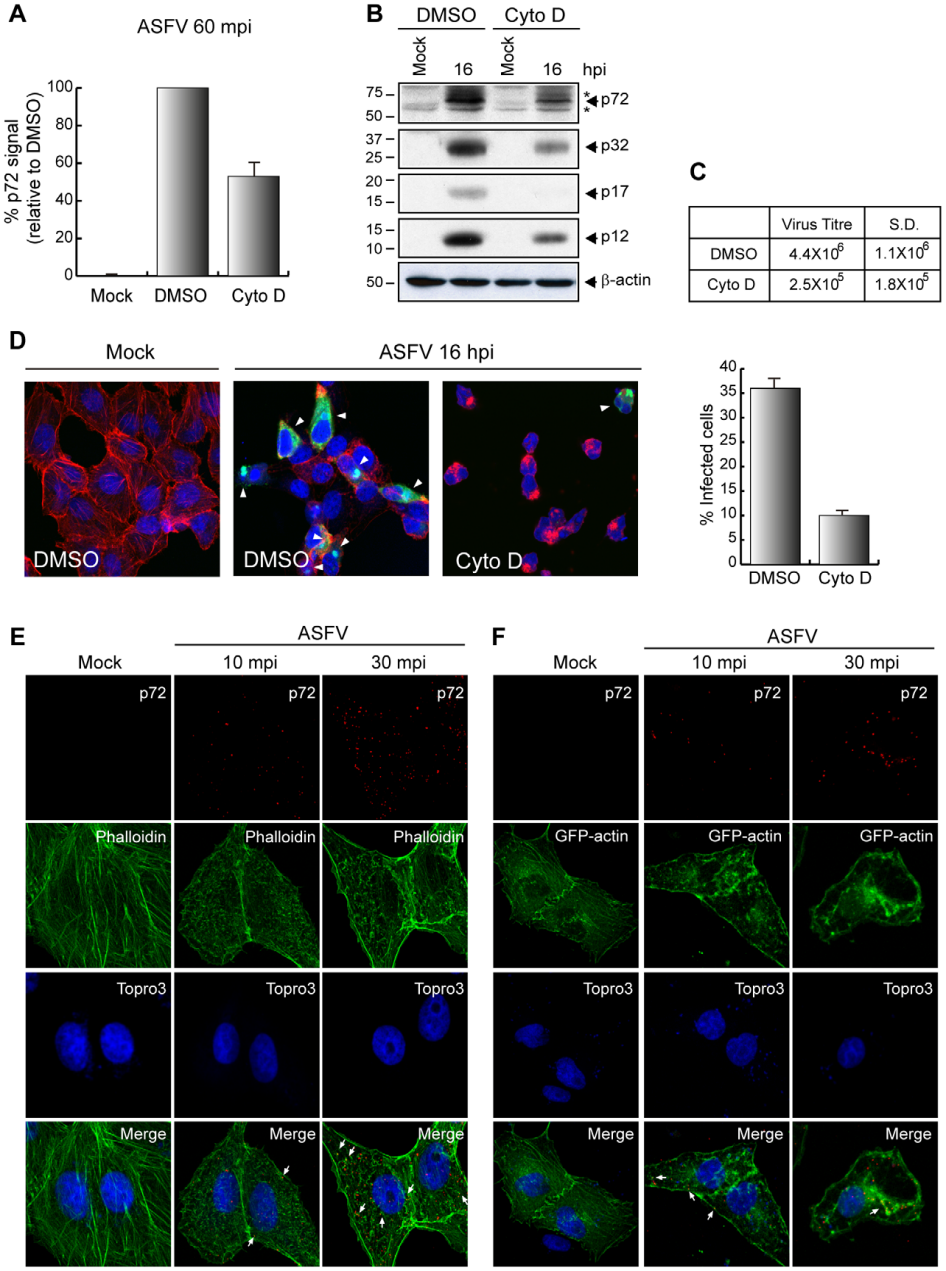
Actin dynamics is important for first steps during ASFV entry in Vero cells. **A–D**) Disruption of actin dynamics reduces the entry of ASFV. **A**) Uptake assays were performed by FACS. Pretreated cells with DMSO or 8 µM Cyto D were infected (MOI 10) for 60 min. Graphic shows percentage of virus entry relative to DMSO control, measured as p72 signal (n = 3, performed in duplicate; mean ±S.D). **B**) Cells were pretreated (4 µM Cyto D) and infected (MOI 1) for 16 h. Equivalent amounts of protein were analyzed by Western blot with an anti-ASFV antibody. β-actin was detected as a load control. **C**) After 48 hpi (MOI 1) supernatants from treated cells (8 µM Cyto D) were recovered and lytic viruses were titrated (n = 3, mean ±S.D). **D**) Development of viral factories (arrowheads) was analyzed by CLSM after treatment (8 µM Cyto D) and infected (MOI 5) for 16 h. Fixed cells were stained with Topro3 (blue), TRITC-phalloidin (red), and anti-p72 (green) to visualize cell nuclei, actin filaments and viral factories, respectively. Images of a mid z-section are shown. The percentage of infected cells of three independent experiments from CLSM images (100 cells per condition) is represented in graphic format (mean ±S.D.). **E–F**) ASFV infection induces rearrangements of the actin cytoskeleton. Cells were infected at a MOI of 50 pfu/cell (E) or transfected with pEGFP-actin for 16 h and then infected (MOI 50). For both, E and F, cells were fixed at indicated times post infection and incubated with Alexa Fluor 488-phalloidin (E), anti-p72 and Topro3 (E and F) to stain actin filaments, viral particles and cell nuclei, respectively. Z-slides images were taken by CLSM and represented as a maximum of z-projection. S.D., standard deviation; Cyto D, Cytochalasin D. ^*^ Unspecific cellular protein detected by the antibody.

To demonstrate whether ASFV depends on actin to enter cells, we used Cyto D, which binds to the positive end of F-actin impairing further addition of G-actin, thus preventing growth of the microfilament [67]. Vero cells were pretreated with Cyto D at a concentration of 8 µM and ASFV uptake (MOI 10) at 60 mpi was next analyzed by FACS. As shown in Figure 4A, the disruption of actin dynamics by the inhibitor reduced ASFV entry in a percentage of about 50%. To assess whether the drug impairs the synthesis of viral proteins, Vero cells were untreated or treated with Cyto D (4 µM) and then infected with Ba71V, MOI 1. After 16 hpi, we used a specific antiserum against both early and late ASFV proteins (generated in our lab), to analyze viral protein expression. As expected, Cyto D treatment importantly reduced both the synthesis of p32, one of the main ASFV early proteins, and the synthesis of p12, p17 and p72, three typical late proteins in the ASFV cycle (Figure 4B). In agreement with this, both virus production and viral factories clearly diminished as shown in Figure 4C and 4D, respectively. However, it is noteworthy that even in the presence of Cyto D, a number of virions seem able to enter the cell and induce a productive infection, thus suggesting that the actin cytoskeleton is involved in ASFV entry and also in successive post-entry steps, as shown in Figure S4B.

To further assess the importance of actin microfilaments in the first steps of ASVF entry, we examined whether ASFV infection causes rearrangements of actin cytoskeleton in Vero cells, by using phalloidin in confocal microscopy experiments. Data are presented in Figure 4E, showing the change of actin pattern after 10 and 30 min after virus uptake at MOI 50. Furthermore, and to reinforce these data, Vero cells were transfected with pEGFP-actin plasmid (kindly gifted by Dr. J. Mercer), and infected with Ba71V, MOI 50. Figure 4F shows the redistribution in aggregates of GFP-actin in transfected Vero cells, which are similar to those observed when endogenous actin was analyzed. Not only that, but also, viral particles (red) are found together with the actin aggregates both in endogenous and ectopically expressed actin.

Since it has been described that blebs and ruffles contain actin, Rac1 and cortactin [23], [68], it is likely that these actin spots correspond to membrane active places where ASFV-induced ruffling should occur, thus suggesting that actin dynamics is a very important factor to ASFV in the host cell to mediate cell-wide plasma membrane ruffling.

Another component of the cytoskeleton that has been reported to be involved in several virus entry processes is the microtubules system, although the importance of microtubules specifically regarding the macropinocytosis pathway is controversial [69]. In respect to ASFV infection, whereas it has been reported that nocodazole (a specific inhibitor of microtubules system [70]) does not affect viral DNA replication [71], a report from Health et al. [72] describes that nocodazole produces a decrease in the expression of p72 and p12 late proteins, but not in the early proteins of ASFV. To investigate whether the microtubule system has a role in ASFV entry, Vero cells were treated with different concentrations of nocodazole and then infected with ASFV at MOI 1. Microtubule disruption had no effect on early viral protein synthesis and barely on late proteins synthesis such as p12 and p72 (Figure S5). Therefore, we conclude that the microtubules system is not likely significant for ASFV entry.

### ASFV induces EGFR and PI3K-Akt pathway activation

Macropinocytosis is typically started by external stimulation. This stimulation is usually associated with growth factors that trigger activation of receptor tyrosine kinases (RTKs). These molecules then activate signaling pathways that induce changes in the dynamics of actin cytoskeleton and disturb plasma membrane [21]. Among them, epidermal growth factor receptor (EGFR) has been connected with actin rearrangement and activation of Rho family GTPases, and its activation is known to trigger macropinocytosis [45], [73].

**Figure 5:**
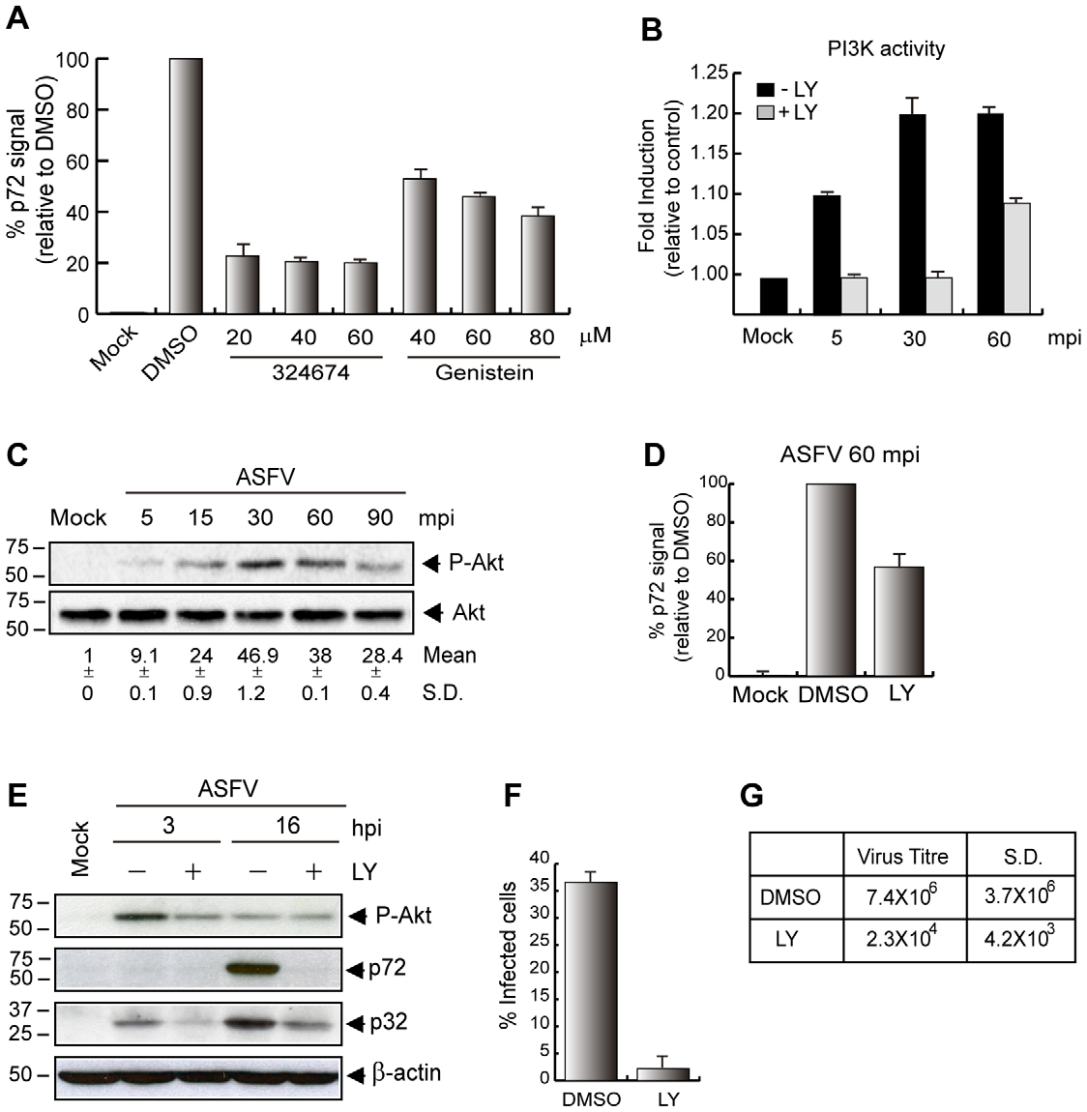
Role of PI3K-Akt, EGFR and tyrosin kinases on ASFV entry and infection in Vero cells. **A**) EGFR and tyrosin kinases are important for ASFV uptake. Virus entry was measured by FACS in cells pretreated with 324674 and Genistein after 60 mpi (MOI 10). Graphic shows percentage of virus entry relative to DMSO control, measured as p72 signal (n = 3, performed in duplicate; mean ±S.D.). **B**) PI3K is activated during ASFV entry. Pretreated cells (DMSO or 40 µM LY) were infected (MOI 10) and PI3K activity was measured by ELISA kit at indicated times post infection. **C**) ASFV infection induces Akt phosphorylation. Cells were infected (MOI 10) and phosphorylation level of Akt was analyzed by immunoblotting with a phospho-Akt Ser473 antibody. Total Akt levels were measured as a control and fold induction was determined by densitometry (mean±S.D). **D**) PI3K is required for ASFV entry. Cells were pretreated (60 µM LY) and infected (MOI 10) for 60 min to analyze ASFV uptake by FACS. Graphic shows percentage of virus entry relative to DMSO control, measured as p72 signal (n = 4, performed in duplicate; mean ±S.D.). **E**) PI3K plays an important role in viral protein synthesis. Cells were pretreated (20 µM LY) and infected (MOI 1) for 3 and 16 h. Viral protein synthesis was analyzed with anti-p32 and anti-p72 polyclonal antibodies by immunoblotting. Phospho-Akt and β-actin were measured as controls. **F**) LY inhibits viral factory development. Infected cells (MOI 5) were pretreated (60 µM LY) and viral factories (stained with anti-p72 at 16 hpi) were analyzed by CLSM. 100 cells per condition were counted and data represented by the graphic (n = 3, mean ±S.D). **G**) Viral production in the presence of LY. Supernatants from treated cells (20 µM LY) were recovered after 48 hpi (MOI 1) and lytic viruses were titrated (n = 3, mean ±S.D). S.D., standard deviations; LY, LY294002.

Besides the membrane perturbations and actin remodeling observed following ASFV uptake, we have found that EGFR activation was essential for ASFV infection, since 324674, the specific inhibitor of this receptor tyrosine kinase [74], efficiently inhibited ASFV uptake in a dose-dependent manner as assessed by FACS experiments in Vero cells. Accordingly, ASFV entry relies on tyrosine kinases activity, as preincubation of the cells with genistein (tyrosine kinase inhibitor [75]) also inhibited ASFV infection (Figure 5A).

The PI3K/PDK1/Akt/mTORC1 pathway regulates vital cellular processes that are important for viral replication and propagation, including cell growth, proliferation, and protein translation [76]. Concerning macropinocytosis, it has been described that PI3K and its effectors induce the formation of lipid structures in ruffles and macropinocytic cups involved in cytoskeleton modulation [77]–[79]. In recent years, it has been reported that several viruses use the PI3K-Akt pathway to support entry into cells and early events of the infection [23], [80].

In order to investigate the importance of this pathway on ASFV entry, we have developed, after different times of ASFV uptake, an ELISA test that directly measures the activity of PI3K by analyzing phosphorylation of its specific substrate PI(4,5)P_2_. The results (Figure 5B), show the increase of substrate phosphorylation from 5 min after virus uptake, reaching a maximum after 30 min of infection. Importantly, the presence of the PI3K inhibitor LY294002 (LY) [81] strongly impaired the kinase activation by the virus.

It has been reported that Akt is the major downstream effector of the PI3K pathway and is commonly used as readout of PI3K activation [82], since Akt phosphorylation has been considered to be a direct consequence of PI3K activation pathway [83]–[85]. To analyze the effect of virus uptake on Akt phosphorylation, Vero cells were serum starved for 4 h and then infected with Ba71V (MOI 10) from 5 to 90 min. Figure 5C shows that Akt is phosphorylated from 5 min after virus uptake, reaching a maximum at 30 min. It has been established that Akt phosphorylation of Thr308 is a direct consequence of PI3K activation pathway [83] while phosphorylation of Ser473 depends on mTORC2 [84], [85]. Since phosphorylation in both residues of Akt is required for its complete activation, we measured the ASFV-induced Akt phosphorylation with two different anti-phospho antibodies. Figure S6 shows that Akt is phosphorylated both in Thr308 and in Ser473 early after ASFV infection, suggesting that ASFV entry fully activates this pathway in the infected cell.

To further investigate whether the PI3K activation observed early during ASFV infection involves mainly upstream steps, we pretreated Vero cells with LY at a concentration of 60 µM. Cells were then infected with Ba71V MOI 10, and the virus uptake was analyzed by FACS at 60 mpi. Figure 5D shows that virus uptake decreased to about 45% in treated Vero cells in respect to DMSO-treated cells, indicating that PI3K activation is involved in the virus entry. Not only that, but we also found that the activation of this kinase has a key role in the consecution of infection since, as shown in Figure 5E, the presence of 20 µM LY severely impairs the synthesis of both ASFV early and late virus proteins. Recently, our group has described that ASFV regulates the cellular machinery of protein synthesis to guarantee the expression of its own proteins [15]. Since it has been reported that one of the main roles of PI3K is regulating the translational machinery through the PI3K-Akt-mTOR pathway [86], the strong effect observed of LY on the ASFV protein synthesis is not surprising (Figure S4C). Finally, and to confirm the role of PI3K on ASFV infection, we performed experiments to analyze the number of cells presenting viral factories in the presence of LY. As shown in Figure 5F, a dramatic decrease of infected cells was observed after 16 hpi (MOI 5) when the infection was performed in the presence of the inhibitor. Similarly, virus production was diminished about 3 logs units by the effect of LY after 48 hpi (Figure 5G).

### ASFV triggers Rac1 activation to enter into the host cells

**Figure 6:**
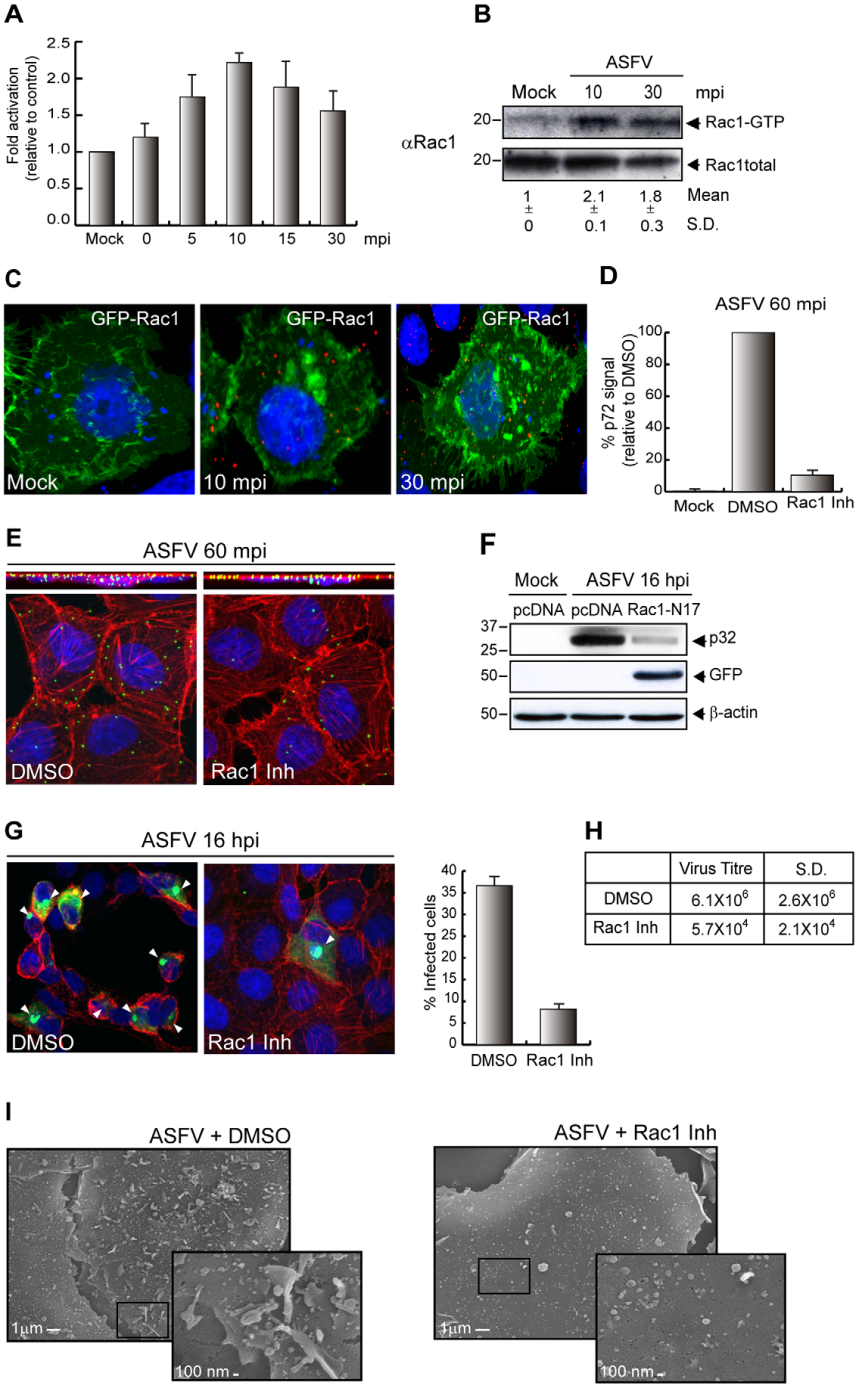
Rac1 plays a critical role in ASFV entry in Vero cells. **A–B**) Activation of Rac1 during ASFV entry. Vero cells were infected (MOI 10) and 0.Rac1 activation was measured by **A**) Kit Activation Assay (n = 3; mean ±S.D.) and **B**) Pak1 PBD-Agarose Beads pull down assay. Fold induction was determined by densitometry (mean ±S.D). **C**) ASFV infection induces clustering of Rac1. Cells were transfected with pEGFP-Rac1, infected (MOI 10) and stained with Topro3 (blue) and anti-p72 (red). Analyzed images by CLSM were represented as a maximum of z-projection. **D–E**) Rac1 inhibitor blocks viral entry. Pretreated cells (200 µM Rac1 inhibitor) were infected (MOI 10) for 60 min. **D**) Graphic shows percentage of virus entry relative to DMSO control, measured as p72 signal analyzed by FACS (n = 3, performed in duplicate; mean ±S.D.). **E**) Cells were incubated with Topro3 (blue), TRITC-phalloidin (red) and anti-p72 (green). Images are represented as a maximum z-projection of x-y plane (bottom panels) and x–z plane (upper panels). **F**) Expression of inactive form of Rac-1 reduces viral infection. Transfected cells with pcDNA or pGFP-Rac1-N17 were infected (MOI 1) for 16 h. Viral protein synthesis was analyzed by immunoblotting with an anti-p32 antibody. GFP and β-actin levels were measured as a control. **G–H**) Rac-1 inhibitor affects ASFV infection. **G**) Viral factory formation was analyzed in pretreated and infected cells (MOI 5) for 16 h. Cells were fixed and stained with Topro3, TRITC-phalloidin and anti-p72. Arrowheads: viral factories. Percentage of the infected cells is represented in left graphic (100 cells/condition; n = 3; mean ±S.D.). **H**) After 48 hpi (MOI 1) supernatants from treated cells were recovered and lytic viruses were titrated (n = 3). **I**) ASFV-induced ruffles are inhibited by Rac1 inhibitor. Cells were pretreated (200 µM Rac1 inhibitor), infected (MOI 50) for 10 min, fixed and analyzed by FESEM. S.D., standard deviations.

Since activation of Rac1-GTPase has been involved in the regulation of macropinocytosis by triggering membrane ruffling in the cell [87], we investigated the activation status of Rac1 during the first steps of ASFV entry in Vero cells. Ba71V was used to synchronously infect cells (MOI 10), and Rac1 activation was measured with the G-LISA activation kit following the manufacturer's instructions. The results showed that Rac1 activation is a very fast and strong event during ASFV entry, reaching a maximum (2.5 fold) at 10 mpi compared to mock-infected cells (Figure 6A). It has been shown that Rac1 controls macropinocytosis by interacting with its specific effectors, the p21-activated kinases (Paks), thus modulating actin cytoskeleton dynamics [88], [89]. It is also known that Rac1 binds and activates Pak1 only under its Rac1-GTP active form. To confirm the results obtained by G-LISA, we further analyzed the Rac1 activation during ASFV entry by performing a pull down assay using Pak1-PBD-Agarose Beads, which carried the PBD-Pak1 ready to bind Rac1-GTP. As shown in Figure 6B, Rac1-GTP was found together with the pulled Pak1-PBD-Agarose Beads after 10 min post ASFV infection, slightly diminishing 30 min after the infection. This result further corroborates that ASFV entry induces the formation of the Rac1 active conformation. Since it has been described that Rac1 is contained in blebs and ruffles [22], [23], [90] and, as shown above, ASFV induces these type of the structures when it infects cells, we next analyzed the localization of Rac1 during the process of ASFV entry. To achieve this, Vero cells were first transfected for 24 h with pEGFP-Rac1 (kindly given by Dr. J. Mercer) and then infected with Ba71V, MOI 10. As shown in Figure 6C, we found clusters of the GTPase as early as 10 min after infection. Accordingly with the experiments shown above, this effect was clearly perceptible at 30 mpi, demonstrating, first, that ASFV infection induces accumulation of active Rac1 in ruffling areas, and second, that this is an event that takes place mainly during ASFV entry.

The effect of Rac1 inhibition on virus uptake was next investigated. Cells were pretreated with 200 µM Rac1 inhibitor [91] and the virus uptake was measured after 60 mpi by FACS analysis, using the specific antibody against the ASFV capsid protein p72, as described in Materials and Methods. Figure 6D shows the dramatic decrease of virus uptake when the infection is performed in the presence of the pharmacologic inhibitor of Rac1. Furthermore, we analyzed the effect on the ASFV uptake in the presence of the inhibitor by CLSM experiments, using the same conditions as above. The images were taken as a maximum z-projection of horizontal and vertical slices. As Figure 6E (bottom panels) indicates, a strong inhibition of virus uptake could be observed in the presence of the Rac1 inhibitor, since the number of ASFV particles in the cell (green) is visibly lower in the presence of the drug. Moreover, and as shown in the upper panels of Figure 6E, virus (green) colocalized (yellow), with cortical actin (red), indicating that the drug immobilizes the virions imbibed into the plasma membrane and impairs their entry into the cell. Separated channels are also shown in Figure S2D.

Alternatively, and to reinforce the role of Rac1 on ASFV infection, we studied the level of ASFV protein synthesis in Vero cells previously transfected with the mutant pGFP-Rac1-N17 (a kind gift from Dr. R. Madrid). The expression of the inactive form of Rac1 strongly inhibited the expression of the ASFV early p32 protein (Figure 6F). As expected, the synthesis of viral late proteins was also affected by treatment with the inhibitor (Figure S7). Not only that, but also, when Rac1 inhibitor was added 60 min after virus addition, the level of viral protein synthesis observed was completely recovered, thus reinforcing the role of Rac1 in virus entry (Figure S4D).

Hence, the role of Rac1 on ASFV morphogenesis and virus production was investigated. To achieve this, Vero cells were treated with the Rac1 inhibitor and then infected during 16 h, MOI 5. Cells were fixed and stained with anti-p72 to visualize the viral factories by CLSM and the percentage of infected cells in the presence or absence of the inhibitor was represented in the graph (Figure 6G). As observed, the number of cells containing ASFV factories decreased about 65% in the presence of Rac1 inhibitor compared to the untreated controls (separate channels are shown in Figure S2E). In line with these results, the viral production at 48 hpi decreased strongly when the activity of Rac1 GTPase was inhibited (Figure 6H).

Finally, since Rac1 has been reported to be an important component of ruffles [22], [23], [90], we have used the Rac1 inhibitor to assess its involvement in the inhibition of these membrane perturbations and therefore, indirectly, the role of ruffles in ASFV uptake. To achieve this, we have performed FESEM assays in Vero cells treated with 200 µM Rac1 inhibitor during 60 min prior to virus addition. As shown in Figure 6I, Rac1 inhibitor strongly decreases the ASFV-induced ruffles, in accordance with the decrease in virus uptake (Figure 6D), viral infection (6G) and virus production (6H) previously observed.

Taken together, these results demonstrate the significant role of Rac1 on ASFV entry.

### Pak1 activation has a key role in ASFV infection

The p21-activated kinase 1 (Pak1), a serine/threonine kinase activated by Rac1 or Cdc42 [89] is one of the most relevant kinases related to several virus entry processes since it is involved in the regulation of cytoskeleton dynamics and is needed during all the stages of macropinocytosis [88], [92], [93]. Among the different residues to be phosphorylated in Pak1 activation, the Thr423 plays a central role because its phosphorylation is necessary for full activation of the kinase [94].

**Figure 7:**
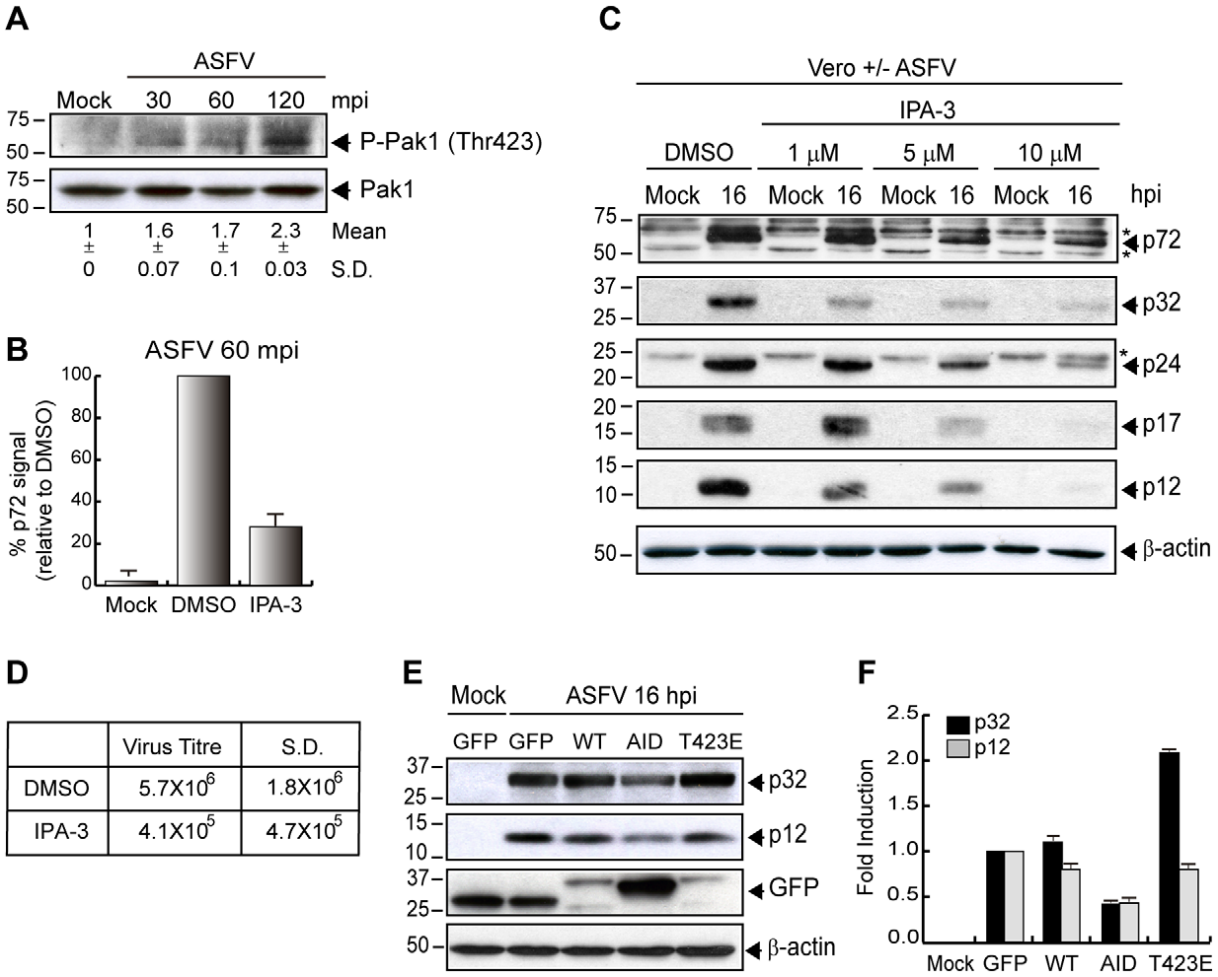
Pak1 is required for ASFV entry in Vero cells. **A**) ASFV activates Pak1 at early times post infection. Cells were infected (MOI 5) and phosphorylation of Pak1 (Thr423) was determined at different times after infection by Western blot. Levels of total Pak1 were measured as a control. Fold induction was determined by densitometry (mean ±S.D). **B–D**) IPA-3 inhibits ASFV entry. **B**) Cells were pretreated with DMSO or 30 µM IPA-3 and infected (MOI 10) for 60 min to analyze ASFV uptake by FACS. The graph shows percentage of virus entry relative to DMSO control, measured as p72 signal (n = 9, performed in duplicate; mean ±S.D.). **C**) Viral protein synthesis was analyzed in infected cells (MOI 1) at 16 hpi in the presence of IPA-3 at the indicated concentrations. Equivalent amounts of protein were analyzed by Western blot with an anti-ASFV antibody. **D**) Supernatants from DMSO or 5 µM IPA-3 treated cells after 48 hpi (MOI 1) were recovered. Lytic viruses were titrated in Vero monolayers and plotted in the table (n = 3). **E–F**) Pak1 mutant reduces ASFV infection. **E**) Vero cells were transfected with pEGFP-Pak1-WT, pEGFP-Pak1-AID (Pak D/N form) and pEGFP-Pak1-T423E (Pak C/A form) for 24 h. Then, cells were infected (MOI 1) for 16 h and viral protein synthesis was analyzed by immunoblotting with an anti-ASFV antibody. GFP expression was measured as a control of transfection. β-actin was detected as a load control. **F**) Fold induction was determined by densitometry and represented in the graphic (mean ±S.D). S.D., standard deviation. ^*^ Unspecific cellular protein detected by the antibody.

To determine whether Pak1 was activated during ASFV entry, we first analyzed the phosphorylation on Thr423 in Vero cells synchronously infected (MOI 5) with Ba71V. At different times post infection, samples were collected and analyzed by immunoblotting using an anti-phospho-Pak1 Thr423 antibody. As early as 30 mpi, phosphorylation of Pak1 could be detected, increasing until 120 mpi (Figure 7A).

IPA-3 has been identified as a direct, noncompetitive and highly selective Pak1 inhibitor. In the presence of IPA-3, Thr423 phosphorylation is inhibited since the Pak1 autoregulatory domain is targeted by the inhibitor [95]. To assess the role of Pak1 activation in ASFV uptake, we measured by FACS analysis the p72 levels detected into the Ba71V-infected Vero cells (MOI 10) after 60 mpi. As shown in Figure 7B, the p72 levels incorporated into the cells in the presence of 30 µM IPA-3 were significantly lower (70%) than those obtained in the absence of the inhibitor. These results indicate that Pak1 activation is involved in the first stages of ASFV entry, since phosphorylation of the kinase occurs at very early times after virus addition, and even more importantly, the uptake of the virus into the host cells is strongly dependent of Pak1 activity.

Apart from the role played by Pak1 in viral entry, the sensitivity of ASFV infection to IPA-3 was investigated in Ba71V-infected Vero cells by Western blot. Using specific antibodies against both early and late ASFV proteins, the effect of the inhibitor from 1 to 10 µM on viral protein synthesis was evaluated. Figure 7C shows the strong dose-dependent IPA-3 inhibition over the most important early (p32) and late proteins (p72, p24, p17 and p12). To reinforce the role of Pak1 in ASFV entry, a similar experiment performed by incubation with IPA-3 during 60 min after virus addition is shown in Figure S4E. These data indicate that the drug is mainly affecting virus entry as it does not induce important inhibition on viral protein synthesis when incubated after virus uptake. Moreover, virus title was reduced 1.5 log units in cells pretreated with 5 µM IPA-3 and then infected with Ba71V (MOI 1) in the presence of the inhibitor during 48 h (Figure 7D).

To corroborate the significant role of Pak1 during ASFV infection, we used different Pak1 constructs affecting Pak1 activation (see Materials and Methods). Vero cells were transfected for 24 h with pEGFP, pEGFP-Pak1-WT, pEGFP-Pak-AID and pEGFP-Pak1-T423E (all of them kindly gifted by Dr. J. Chernoff) and infected for 16 h with ASFV at a MOI of 1 pfu/cell. As shown in Figure 7E, the constructs containing the Pak1 autoinhibitory domain (AID) inhibited p12 and p32 viral protein expression, whereas cells transfected with wild type (WT) form showed the same protein levels than infected control cells. It is noteworthy that constitutively active Pak1 construction T423E (even although it was only shortly expressed in the transient transfection process) induced a remarkable enhancement on the expression of the ASFV early protein p32, indicating that increasing Pak1 activity intensifies the early protein synthesis, probably due to its effect on virus entry. Numeric values of these data are shown in Figure 7F.

These data, together with those of Rac1 activation explained above, strongly supports our hypothesis of ASFV triggering the Rac1-Pak1 pathway during the virus entry.

### Role of dynamin and clathrin during ASFV entry and infection

Dynamin is a cellular essential GTPase which plays an important role in cellular membrane fission during vesicle formation [96]. It is likely involved in Rac1 localization and function, since it has been shown that Rac1-dependent macropinocytosis is blocked by the dynamin-2 (DynK44A) dominant-negative [39].

**Figure 8:**
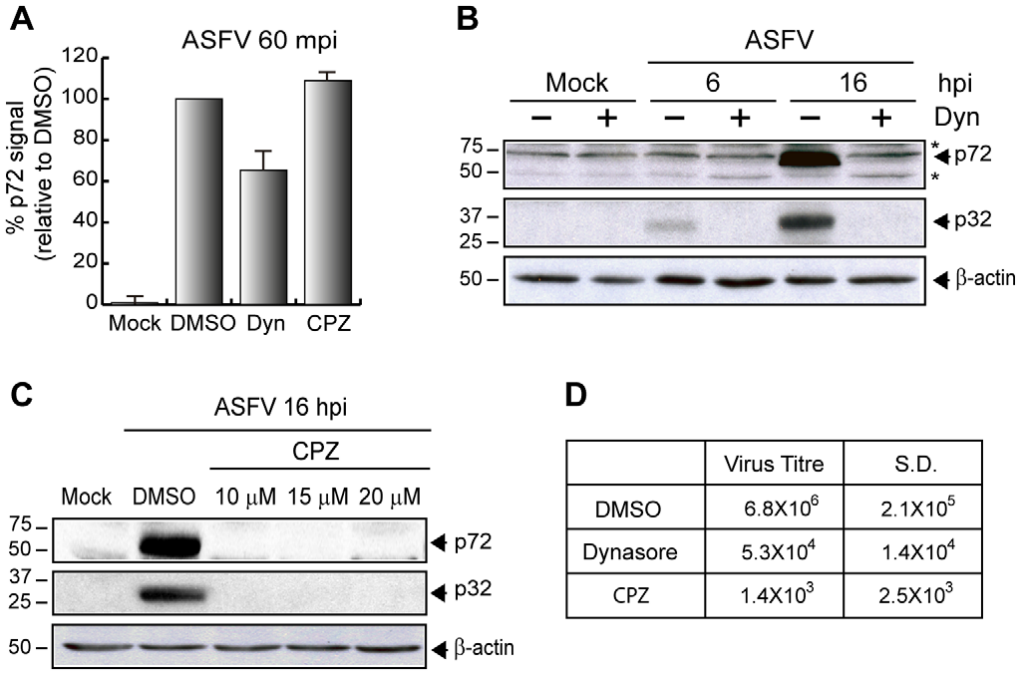
ASFV entry is independent of clathrin-mediated endocytic pathway. **A**) ASFV entry is partially inhibited by Dynasore but not by Chlorpromazine. Pretreated Vero cells with DMSO, 100 µM Dynasore (Dyn) and 20 µM Chlorpromazine (CPZ) were infected (MOI 10) for 60 min. Infected cells were analyzed by FACS and the graph shows the percentage of virus entry relative to DMSO control, measured as p72 signal. Bars represent the mean of three independent experiments (mean ±S.D., performed in duplicate). **B–C**) Dynamin and Clathrin are important for infection progress. Synthesis of viral proteins was measured in infected Vero cells (MOI 1) in the presence of Dyn (B) and CPZ (C) at 6 and/or 16 hpi at the indicated concentrations by Western blot with an anti-ASFV antibody. β-actin was detected as a load control. **D**) Viral production in the presence of Dynasore and Chlorpromazine. After 48 hpi (MOI 1) supernatants from DMSO, Dyn (100 µM) and CPZ (20 µM) treated cells were recovered. Lytic viruses were titrated in Vero monolayers and plotted in the table (n = 3). S.D., standard deviations; Dyn, Dynasore; CPZ, Chlorpromazine. ^*^Unspecific cellular protein detected by the antibody.

Since, as we demonstrated above, Rac1 is important to ASFV entry, we have analyzed whether dynamin-2 pathway plays a role either in ASFV entry or infection. To achieve this, we first investigated the effect of Dynasore (Dyn), a reversible inhibitor of GTPases activity [97], over ASFV uptake. After 60 min of pretreatment with 100 µM Dyn, Vero cells were infected with Ba71V at MOI 10 and virus uptake was measured by FACS using the specific antibody against the capsid viral protein p72. The result showed that treatment with Dyn partially inhibited virus uptake (35%) (Figure 8A). A higher effect of the inhibitor on ASFV entry could not be found by using different experimental conditions (data not shown), further indicating the partial involvement of dynamine in virus uptake. Moreover, the role of clathrin-mediated endocytosis was examined in parallel using Chlorpromazine (CPZ), which inhibits the assembly of coated pits at the plasma membrane and is considered a specific inhibitor of clathrin-mediated endocytosis [98]. Using parallel experimental conditions, and in contrast with the data obtained after treatment with Dyn, we observed that the virus uptake was not likely affected in the presence of 20 µM CPZ (Figure 8A). These data indicate that whereas dynamine is to some extent involved in ASFV entry in accordance with its role in macropinocytosis [39], clathrin is not related to ASFV uptake in Vero cells.

In order to investigate whether other steps downstream ASFV entry were affected by Dyn and CPZ, Vero cells were separately pretreated with the inhibitors, and then infected with ASFV (MOI 1). At the indicated times after infection, the synthesis of both early and late ASFV proteins was analyzed by Western blot. The treatment with 100 µM Dyn strongly inhibited p72 and p32 expression from early times post infection (Figure 8B), consequently indicating that dynamine is required for ASFV both early and late infection course. As Figure 8C shows, CPZ had a similar effect to Dyn both on ASFV early and late protein synthesis, in concordance with the data from Hernaez et al. [38], in which the expression of the viral protein p32 depends on clathrin function. Higher amounts of CPZ could result in an inhibition of p72, but this effect is likely due to the cytotoxic effect of the drug, as reported in Table S1. Taken together, our data showed that whereas the effect of Dyn on viral protein synthesis is probably due to dynamine participation on ASFV entry events, the clathrin inhibition does not involve virus uptake, but only viral protein synthesis, thus indicating a role for clathrin function merely in post entry events. Future experiments are planned to more specifically study which are the ASFV post entry events regulated by clathrin.

Finally, and as expected, both inhibitors had an important effect on viral production measured after 48 hpi (MOI 1) in Vero cells (Figure 8D).

## Discussion

Endocytosis constitutes an efficient way for viruses to cross the physical barrier represented by the plasma membrane and to pass through the underlying cortical matrix. Knowledge of the specific pathway of virus entry and of the precise mechanisms regulating is key to understand viral pathogenesis, since virus entry into host cell is the first major step in infection. Whereas there is ample evidence showing that ASFV enters cells through endocytosis in a pH-dependent manner and that saturable binding-sites on the plasma membrane mediate the productive entry of the virus into Vero cells and swine macrophages [33], [34], the specific endocytic and signaling pathways used by the virus are largely unknown.

In this report, by combining different and independent approaches, we have achieved an exhaustive analysis of the ASFV endocytic pathway. We have obtained a precise picture of how ASFV enters the cell and have identified the main cellular proteins required. Careful assessment of specificity and functionality of each pathway was performed and correlated with infection and virus uptake.

Many recent reports have shown that viruses can directly use macropinocytosis as an endocytic way for productive infection [21], [23]–[29], and also to promote the penetration of viral particles that enter by other endocytic mechanisms [31], [32]. Macropinocytosis activation is related to significant cell-wide membrane ruffling mediated by activation of actin filaments. These structures may have different shapes: lamellipodia, circular-shaped membrane extrusions (ruffles) or large membrane extrusions in form of blebs.

Here we have illustrated by FESEM that ASFV strain Ba71V induced prominent membrane protrusions compatible with ruffles after 10 mpi. Transmission electron microscopy images further support this result by showing that ASF virions internalize adjacent to retracting ruffles, likely indicating uptake of viral particles occurs as part of the macropinocytic process. Not only that, but also, we found that inhibition of Rac1, an important component of ruffles, importantly impaired the ASFV uptake, thus involving the formation of these membrane perturbations in virus entry.

Moreover, and in parallel to the data obtained in Vero cells, we found that the E70 virulent strain induced a type of membrane protrusion similar to blebs a few minutes after the infection of the swine macrophage line IPAM. This last result is important, since macrophages are probably the natural target cell of the infection in vivo and suggests that different macropinocytic programs can be used by different ASFV strains, as has been published for other virus as Vaccinia [45]. Because of this, we have carefully characterized these structures. First, we showed the inhibition of virus entry with different doses of blebbistatin, and second we demonstrated that Rock1 (a marker of blebs [52]) colocalized with virus particles on blebs in IPAM cells from 30 min after virus uptake.

Apart from characteristic membrane perturbations, macropinocytosis is also distinguished from other entry pathways by features that include actin-dependent structural changes in the plasma membrane, regulation by PI3K, PKC, Rho family GTPases, Na^+^/H^+^ exchangers, Pak1, as well as ligand-induced upregulation of fluid phase uptake. In this regard, our work demonstrates that EIPA, a potent and specific inhibitor of the Na^+^/H^+^ exchanger [23], [53], [54], [99], severely impairs ASFV infection and entry. By using FACS analysis we found that EIPA treatment caused a significant dose-dependent manner reduction (more than 60%) in the uptake of ASFV infective particles. Confocal microscopy analysis also revealed that there was an evident drop in virus particles incorporated into the cells incubated with EIPA. It is important to note that macropinocytosis is the only endocytic pathway susceptible to the inhibition of the Na^+^/H^+^ exchangers. Thus, these results strongly indicate the involvement of macropinocytosis in ASFV virus entry.

Actin plays a central role in formation and trafficking of macropinosomes. Cyto D, which binds to the positive end of F-actin (impairing further addition of G-actin and preventing the growth of the microfilament [67]), reduced ASFV entry by approximately 50% and inhibited the synthesis of both early and late viral proteins, together with viral morphogenesis. However, it is remarkable that virions that escape from the action of Cyto D induce a productive infection, thus suggesting that actin cytoskeleton is mainly involved in ASFV entry, although it could have a role in successive post-entry steps.

Corroborating this hypothesis, we have observed that ASFV infection causes rearrangements of endogenous actin cytoskeleton in Vero cells as early as 10 min post infection. These data were reinforced by overexpression of GFP-actin that was concentrated in aggregates in virus-infected cells. Together, these data provide evidence for a role of actin in ASFV entry and suggest that the virus can actively promote localized actin remodeling to facilitate its uptake through macropinocytosis or a similar mechanism.

The first reports describing the endocytic entry of viruses into their host cells presumed that incoming viruses took advantage of ongoing cellular endocytosis processes [16]. However, it is now clear that several viruses are not only passive cargo but activate their own endocytic uptake by eliciting cellular signaling pathways. The activation of these pathways significantly depends on the interaction of the virus with cellular receptors specific to the type and activation status of the host cell [100], [101]. ASFV, as Vaccinia virus [21], [45], seems to belong to the viruses that actively trigger their endocytic internalization. In this respect, we have found that entry of ASFV is dependent on signaling through tyrosine kinases as EGFR, and activation of PI3K together with Rho-GTPases as Rac1, which have been all described to be important regulators of macropinocytosis [69].

Concerning the function of the PI3K pathway, activation of this kinase early after virus uptake was confirmed by analyzing the phosphorylation of its specific substrate PI(4,5)P_2_. Also, phosphorylation of both residues Thr308 and Ser473 of Akt was observed early after ASFV infection. Besides, pretreatment of Vero cells with the specific PI3K pharmacological inhibitor LY strongly inhibited virus uptake at 60 mpi. Not only that, but we also found that the activation of this kinase has an important role in the infection, since the presence of LY severely impairs the synthesis of both ASFV early and late virus proteins. In this regard, our group has recently described [15] that ASFV uses the cellular machinery of protein synthesis to express its own proteins. Since it has been reported that one of the main roles of PI3K is to regulate the translational machinery through the AKT-mTOR pathway [86], the strong effect observed of LY on ASFV protein synthesis is very much expected.

We have also demonstrated that Rac1, a regulatory guanosine triphosphatase of Pak1, was activated during ASFV entry. Rac1 protein belongs to the Rho family of small guanosine triphosphatases, a subgroup of the Ras superfamily of GTPases [102]. In the last years, several viruses have been described to target Rho-GTPases activation to enter the host cells, such as Vaccinia virus [23], [45], Ebola virus [80], Echovirus [92] or Adenoviruses type 2 [103], among others. Through interaction with its specific effector Pak1, Rac1 modulates actin cytoskeleton dynamics and controls macropinocytosis [88], [89]. Consistent with the data reported by Mercer and Helenius, 2008 [23], showing that active Rac1 is contained in virus-induced membrane perturations, our results show that ASFV induces clusters of this GTPase as early as 10 min after infection. Hence, Rac1 accumulates in ruffling areas very early during the process of ASFV entry, suggesting that ASFV targets Rac1 to entry in host cells. In agreement with this hypothesis, a strong inhibition of virus uptake, in parallel with ruffle formation, was observed in the presence of the Rac1 inhibitor. Moreover, by performing CLSM experiments, we showed that the drug immobilized the virus particles imbibed into the plasma membrane, thus impairing their entry into the cell. Taken together, these results demonstrate the significant role of Rac1 on ASFV entry. Our data strongly contrasts with a recent study [104], which reported that, although Rac1 is activated by ASFV infection, it is not involved in either ASFV entry or viral protein synthesis. In that study by Quetglas et al. [104], Rac1 would be responsible of a downstream process that only affected viral production. The discrepancies about the role of Rac1 in ASFV entry and infection might be explained by the fact that the Rac1 inhibitor concentration used does not match with the amounts usually employed to analyze the role of Rac1 in virus uptake [80], and it is likely too low to disturb ASFV entry or viral protein synthesis. Moreover, confocal microscopy images to measure ASFV uptake were taken as mid z-section, in contrast to our procedure that includes several z-sections that allow us to count the total virus particles inside the cells. Finally, important information regarding the effect of the dominant-negative Rac1-N17 on viral protein synthesis were not shown in that study, in contrast to our results described in Figure 6F. Therefore, the limitations of that work [104] make it difficult to reach any conclusions about the function of Rac1 on ASFV entry and infection. Furthermore, in support of our data, we should note that we have found an important role for Pak1 in Ba71V entry in Vero cells. Pak1 is a serine/threonine kinase activated by Rac1 or Cdc42 involved in the regulation of cytoskeleton dynamics and needed during all stages of macropinocytosis [88], [93], [105]. Our results indicate that Pak1 activation is involved in the first steps of ASFV entry, since phosphorylation of the kinase occurs at very early times after virus addition, and even more importantly, the uptake of the virus into the host cells is strongly dependent of Pak1 activity. However, our preliminary studies using the E70 strain did not show a clear effect of the Pak1-specific inhibitor IPA-3 on the synthesis of ASFV proteins (data not shown), either in IPAM or in alveolar swine macrophages. These data suggest that ASFV may activate other different pathways in macrophages or that IPA-3 cannot be efficient enough to inhibit Pak1 if this kinase is constitutively activated in these cells [106], [107]. Nevertheless, the synthesis of viral proteins was strongly inhibited in macrophages after EIPA and LY treatments, indicating that Na^+^/H^+^ exchangers and the PI3K pathway are involved in macropinocytosis-mediated ASFV entry into these cells (Figure S8).

In conclusion, the involvement of the EGFR and PI3K, the nature of the signaling pathway, the involvement of Rac1, Pak1 and Na^+^/H^+^ exchangers, and the actin-cytoskeleton rearrangements, all support a macropinocytosis-driven endocytic process for ASFV entry. In addition, ASFV caused significant induction of dextran uptake (a specific fluid phase marker of macropinocytosis), and colocalization of the internalized ASF virus particles with dextran was also observed.

The ASFV genome encodes several glycoproteins [108], whose role in host-cell binding and entry has not yet been described. However, it has been shown that glycoproteins and lipids are required for several virus binding and entry steps to the host cells [23], [109], [110]. It has been also reported that cellular partners that bind to specific regions of viral glycoproteins translocate from intracellular compartments to regulate the susceptibility of different cells to the infection [111]. These kinds of mechanisms could explain the differences found among ASFV viral isolates and their ability to infect different host cells. Future experiments are planned to study the role of both ASFV glycolipids and the putative host partners involved in the mechanisms of ASFV entry and infection of different cell populations.

Dynamin is a large GTPase that is involved in scission of newly-formed endocytic vesicles at the plasma membrane [112]–[114]. Although we have shown that dynasore partially inhibits virus entry, we have found no evidence for a role of clathrin in ASFV entry despite the use of multiple approaches. The fact that in our hands dynamin was only partially involved in ASFV entry further ruled out roles for clathrin or caveolae-mediated pathways, as both require dynamin activity. Therefore, our data contrast with a recent study concluding that clathrin-mediated endocytosis is the major entry pathway for ASFV [38]. The key concern about the conclusion of this work is that virus entry is merely measured by the synthesis of ASFV early proteins in the presence of chlorpromazine, and not by specific analysis of virus uptake. Moreover, it is important to note that whereas chlorpromazine disrupts clathrin-coated pits, it may also interfere with biogenesis of large intracellular vesicles such as phagosomes and macropinosomes [115].

Here, by combining different and separate strategies we have carried out a precise analysis of each key endocytic pathway concerned, obtaining, for the first time, a relatively complete description of the mechanism by which ASFV enters into a cell, including identification of several cellular molecules and routes. We have carefully evaluated the specificity and functionality of each pathway and correlated them with virus uptake and infection. Two different strains of ASFV, the virulent E70 and the virulent Ba71V, adapted to growth in Vero cells, have been used to study the virus entry mechanism either in swine macrophages or Vero, respectively.

Several drugs were used to inhibit pathways, but specificity was evaluated by testing the function of the main pathways after treatment. Furthermore, highly specific dominant-negative mutants were used to confirm the data obtained by pharmacological inhibitors. More importantly, all throughout this work either a FACS-based or a confocal sensitive virus entry assays were used in discriminating blockage in virus entry versus blockage in downstream steps of the infection cycle. This is particularly relevant when using drugs that frequently affect multiple cellular functions in addition to specific entry.

Overall, our data provide strong evidence that ASFV entry takes place by a process closely related to macropinocytosis, adding new and valuable information regarding endocytosis mechanisms in the context of ASFV entry (plotted in Table 1).

**Table 1:** Comparison of cellular factors and processes involved in ASFV entry/infection.

Cellular factors	Perturbants/inhibitors	Required for ASFV entry	Required for ASFV infection
Na^+^/H^+^ exchangers	EIPA	Yes	Yes
Actin	Cytochalasin D	Yes	Yes/No
Myosin II	Blebbistatin	Yes	Yes/No
EGFR	EGFR inhibitor	Yes	N/D
PI3K	LY294002	Yes	Yes
Rac1	Rac1 inhibitor, Rac1-N17	Yes	No
Pak1	IPA-3, Pak1-AID	Yes	No
Tyrosine Kinases	Genistein	Yes	N/D
Dynamin-2	Dynasore	Yes	Yes
Clathrin	Chlorpromazine	No	Yes
Microtubules	Nocodazole	No	No
Vacuolar acidification	Chloroquine, NH_4_Cl	No	Yes [36]
Cholesterol	MβCD	Yes [116]	Yes [116]

N/D, designated no data.

The evidence presented demonstrates for the first time, that ASFV utilizes a macropinocytosis-like pathway as the primary means of entry into IPAM and Vero cells. However, we cannot state that virus entry occurs exclusively by this pathway, especially in swine macrophages. But our data clearly show that its disruption blocks the greater part of infection and particle uptake. Our work also indicates that clathrin-mediated endocytosis plays at most a minor role in ASFV entry. However, and in accordance with the data of Hernaez et al. [38], we found that CPZ diminishes both ASFV early and late protein synthesis, together with viral production. Thus, our data demonstrate a role for clathrin function merely in post entry events.

A strong hazard of ASFV dissemination from Sardinia and Caucasian areas to EU countries has recently appeared, thus making the progress of knowledge and tools for protection against this virus urgent. Infection by ASFV is characterized by the absence of a neutralizing immune response, which has so far hampered the development of a conventional vaccine. Therefore, our findings are relevant as they not only provide a detailed understanding of ASFV entry mechanism, but also identify novel cellular factors that may provide new potential targets for therapies against this virus. In parallel, further studies are planned to characterize viral factors that may interact with components of the macropinocytosis pathway, probably useful for vaccine development.

## Supporting Information

Figure S1 **Specificity of p72 antibody and analysis of the ASFV infection.** **A**) Distribution of the p72 protein in the virus particle. Purified virus was treated with different buffers as explained in Materials and Methods. The supernatant (SP) and pellet (P) of the different treatments was analyzed by immunoblotting and p72 protein was detected with a monoclonal antibody (17LD3). **B**) The monoclonal antibody 17LD3 recognizes the viral particles bound to the cell surface. Viral adsorption to cells was allowed for 90 min at 4°C at a MOI of 10 pfu/cell. Sixty min after virus addition, cells were stained for 30 min with 594-WGA to stain the edge of plasma membrane. Cells were stained with anti-p72 monoclonal antibody without permeabilization and fixed finally with paraformaldehide. Images were analyzed by CLSM and represented as a mid z-section. **C**) Monoclonal anti-p72 antibody 17LD3 is a useful tool to follow the infection at early and late times post infection. Vero cells were mock-infected or infected synchronously for 60 min or 16 h at a MOI of 10 pfu/cell and 5 pfu/cell, respectively. At indicated times post infection the cells were fixed with paraformaldehide, permeabilized and stained with Topro3 (blue), TRITC-phalloidin (red) and monoclonal anti-p72 (17LD3) (green) to stain cell nuclei, actin filaments and viral particles (middle panels) or viral factory (bottom panels, arrowheads), respectively. Images were taken by CLSM and represented as a mid z-section.
(TIF)

Figure S2 **Separate channels of CLSM experiments.** **A–E**) Vero cells were pretreated with DMSO or different pharmacological inhibitors and infected with Ba71V for 60 min or 16 h, as indicated in the principal figure legends. The virus uptake or viral factory formation was analyzed by CLSM staining the cell nuclei with Topro3 (blue), actin filaments with TRITC-phalloidin (red) and the virus particles or viral factories with anti-p72 antibody (green). Images were taken by CLSM and represented as a mid z-section or maximum z-projection as indicated. A) Figure 3B; B) Figure 3D; C) Figure 4D; D) Figure 6E; E) Figure 6G. Cyto D, Cytochalasin D; Rac1 Inh, Rac1 inhibitor.
(TIF)

Figure S3 **Effect of macropinocytosis inhibitors on ASFV uptake.** Vero cells were pretreated with DMSO or different pharmacological inhibitors for 60 min at 37°C as follows: 60 µM EIPA, 8 µM Cyto D, 60 µM LY, 200 µM Rac1 Inh and 30 µM IPA-3. Cells were synchronously infected (MOI 10) for 60 min in the presence of the drugs, fixed and stained with Topro3 (blue), phalloidin (red) and anti-p72 (green). Images were taken by CLSM and represented as a maximum z-projection of horizontal slices (x–y plane). The LSM images were imported to Image J program and the number of virus particles inside the cells was automatically counted with a Macro algorithm in which threshold Intermodes was used to define a single virus particle in the cell. The graph shows percentage of virus inside the cells relative to DMSO control of the three independent experiments (mean ±S.D.). S.D., standard deviations; Cyto D, Cytochalasin D; LY, LY294002; Rac1 Inh, Rac1 inhibitor.
(TIF)

Figure S4 **Effect of macropinocytosis inhibitors on virus entry and post entry steps.** Vero cells were treated with 20 µM EIPA (A), 4 µM Cyto D (B), 20 µM LY (C), 200 µM Rac1 Inh (D) and 10 µM IPA-3 (E) for 60 min before the virus addition (Pre-treatment, Pre), or 60 min after virus addition (Post-treatment, Post), and viral infection was allowed in the presence of the drugs at 37°C, in each case. After 16 h, the cells were lysed in RIPA modified buffer and the viral proteins were analyzed by Western blot with an anti-ASFV antibody. β-actin was detected as a load control. Fold induction was determined by densitometry and represented in the graphics below (mean ±S.D.) Cyto D, Cytochalasin D; LY, LY294002; Rac1 Inh, Rac1 inhibitor. ^*^ Unspecific cellular protein detected by the antibody.
(TIF)

Figure S5 **ASFV entry is not dependent on microtubule system.** Vero cells were treated with nocodazole at indicated concentrations and infected with Ba71V (MOI 1) for 16 h. Viral protein synthesis was analyzed by Western blot with an anti-ASFV antibody. β-actin was detected as a load control. ^*^ Unspecific cellular protein detected by the antibody.
(TIF)

Figure S6 **ASFV infection induces Akt phosphorylation at early time post infection.** Vero cells were asynchronously infected (MOI 5) and solubilised in RIPA buffer at the indicated times post infection. Equivalent amounts of protein were analyzed by immunolotting and the phosphorylation level of Akt was analyzed by using specific antibodies against phospho-Akt Ser473 and phospho-Akt Thr308. Levels of total Akt were measured as a control.
(TIF)

Figure S7 **Rac1 inhibitor effect on viral proteins synthesis.** Vero cells were treated with 200 µM Rac1 inhibitor and infected with Ba71V (MOI 1) for 16 h. Samples were solubilised in RIPA buffer and equivalent amounts of protein were analyzed by Western blot with an anti-ASFV antibody. β-actin was detected as a load control. ^*^ Unspecific cellular protein detected by the antibody.
(TIF)

Figure S8 **Factors involved in macropinocytosis during the infection in IPAM cells.** IPAM cells were treated with EIPA and LY (20 µM both) and infected with the isolate E70 during 42 h. Viral protein synthesis was analyzed by Western blot with an anti-ASFV antibody. β-actin was detected as a load control. ^*^ Unspecific cellular protein detected by the antibody.
(TIF)

Table S1 **Citotoxicity of pharmacological inhibitors.** Cells were treated with different pharmacological inhibitors for 3 or 18 hours in serum free medium. Cells were detached with Trypsin-EDTA and stained with Trypan Blue. Percentage of dead cells after treatment is calculated as a number of dead cells relative to live cells. N/D, designated no data.
(TIF)

Video S1 ***In vivo*** **Mock-infected Vero cells.** Vero cells were serum-starved for 24 h and medium without ASFV was added for 90 min at 4°C. Images were collected for 30 min with an Orca R2 digital camera on a widefield microscope with controlled environmental chamber (temperature 37°C and 5% CO_2_ humidified atmosphere). Images were captured at a resolution of 1344×1024 pixels using a 20×, 0.40 NA objective with a 1.6× magnification-changer.
(AVI)

Video S2 ***In vivo*** **ruffle induction in Vero cells by ASFV.** Vero cells were serum-starved for 24 h and ASFV binding was allowed for 90 min at 4°C at MOI 100. After binding, images were collected for 30 min with an Orca R2 digital camera on a widefield microscope with controlled environmental chamber (temperature 37°C and 5% CO_2_ humidified atmosphere). Images were captured at a resolution of 1344×1024 pixels using a 20×, 0.40 NA objective with a 1.6× magnification-changer.
(AVI)
