# Supplementary figures and images for: African Swine Fever Virus Uses Macropinocytosis to Enter Host Cells
Source: PLoS Pathog. 2012 Jun 14;8(6):e1002754. doi: 10.1371/journal.ppat.1002754 (PMC3375293; doi:10.1371/journal.ppat.1002754)

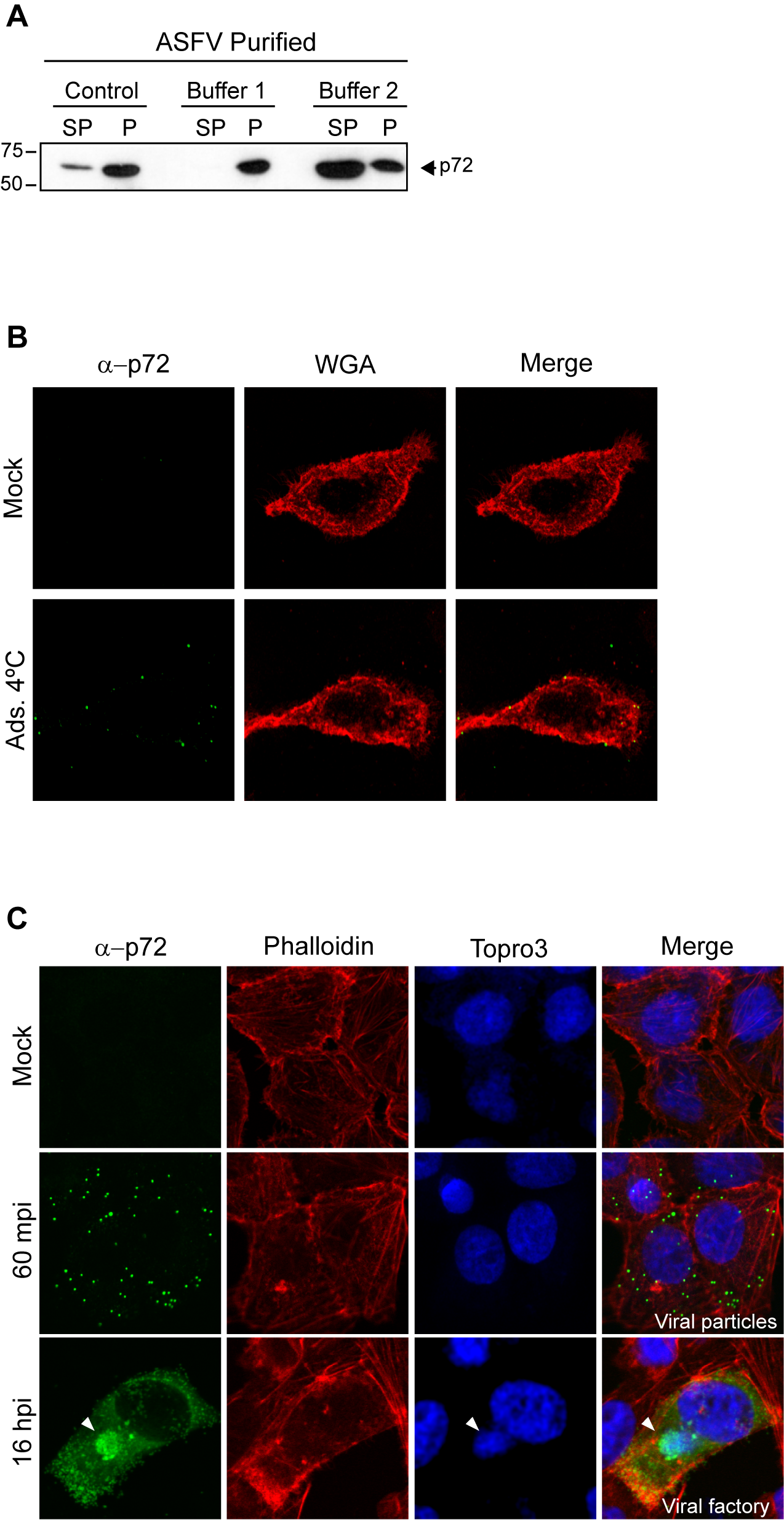

Supplement: Figure S1 — Specificity of p72 antibody and analysis of the ASFV infection. A) Distribution of the p72 protein in the virus particle. Purified virus was treated with different buffers as explained in Materials and Methods. The supernatant (SP) and pellet (P) of the different treatments was analyzed by immunoblotting and p72 protein was detected with a monoclonal antibody (17LD3). B) The monoclonal antibody 17LD3 recognizes the viral particles bound to the cell surface. Viral adsorption to cells was allowed for 90 min at 4°C at a MOI of 10 pfu/cell. Sixty min after virus addition, cells were stained for 30 min with 594-WGA to stain the edge of plasma membrane. Cells were stained with anti-p72 monoclonal antibody without permeabilization and fixed finally with paraformaldehide. Images were analyzed by CLSM and represented as a mid z-section. C) Monoclonal anti-p72 antibody 17LD3 is a useful tool to follow the infection at early and late times post infection. Vero cells were mock-infected or infected synchronously for 60 min or 16 h at a MOI of 10 pfu/cell and 5 pfu/cell, respectively. At indicated times post infection the cells were fixed with paraformaldehide, permeabilized and stained with Topro3 (blue), TRITC-phalloidin (red) and monoclonal anti-p72 (17LD3) (green) to stain cell nuclei, actin filaments and viral particles (middle panels) or viral factory (bottom panels, arrowheads), respectively. Images were taken by CLSM and represented as a mid z-section. (TIF) [file ppat.1002754.s001.tif]

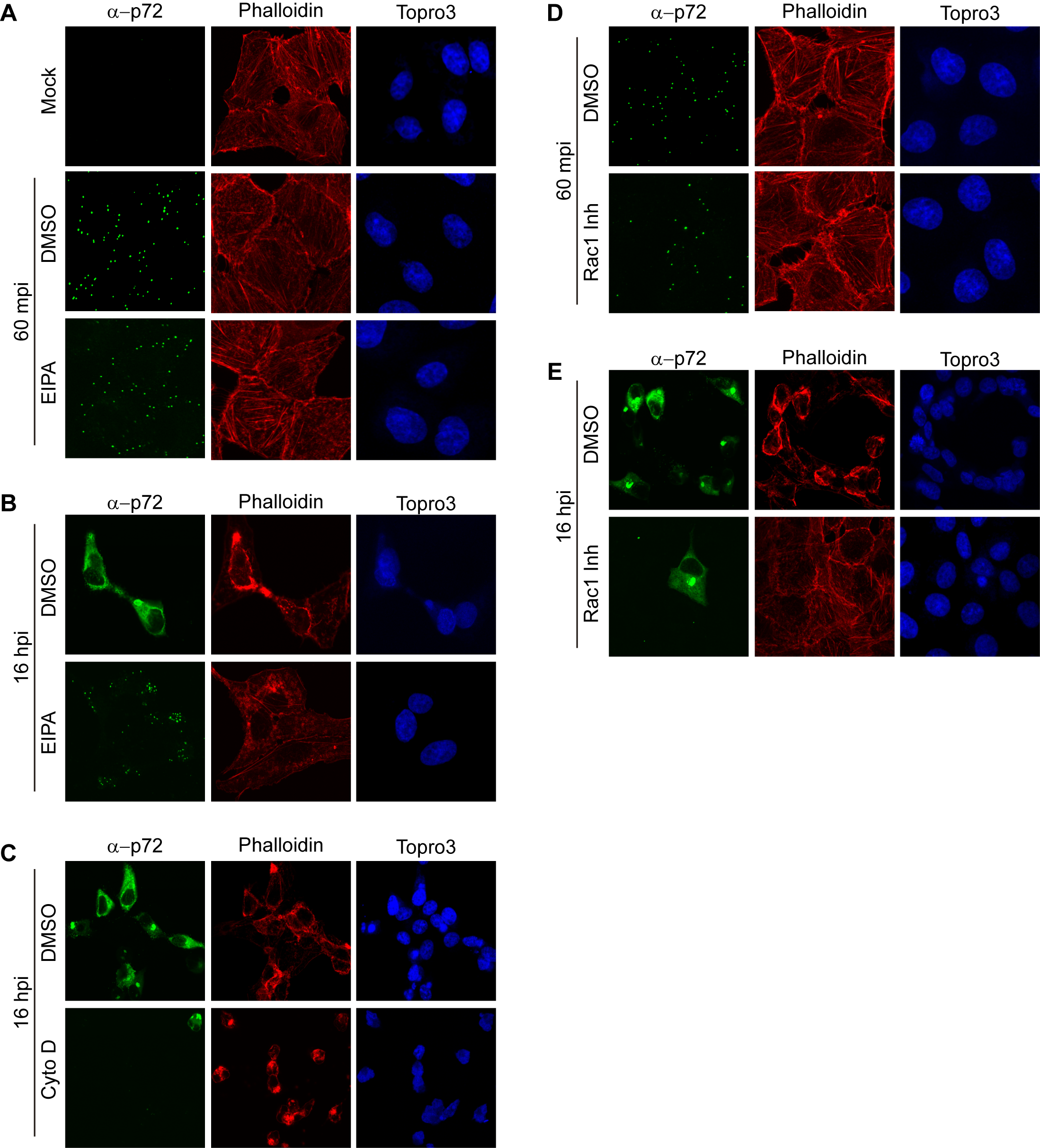

Supplement: Figure S2 — Separate channels of CLSM experiments. A–E) Vero cells were pretreated with DMSO or different pharmacological inhibitors and infected with Ba71V for 60 min or 16 h, as indicated in the principal figure legends. The virus uptake or viral factory formation was analyzed by CLSM staining the cell nuclei with Topro3 (blue), actin filaments with TRITC-phalloidin (red) and the virus particles or viral factories with anti-p72 antibody (green). Images were taken by CLSM and represented as a mid z-section or maximum z-projection as indicated. A) Figure 3B; B) Figure 3D; C) Figure 4D; D) Figure 6E; E) Figure 6G. Cyto D, Cytochalasin D; Rac1 Inh, Rac1 inhibitor. (TIF) [file ppat.1002754.s002.tif]

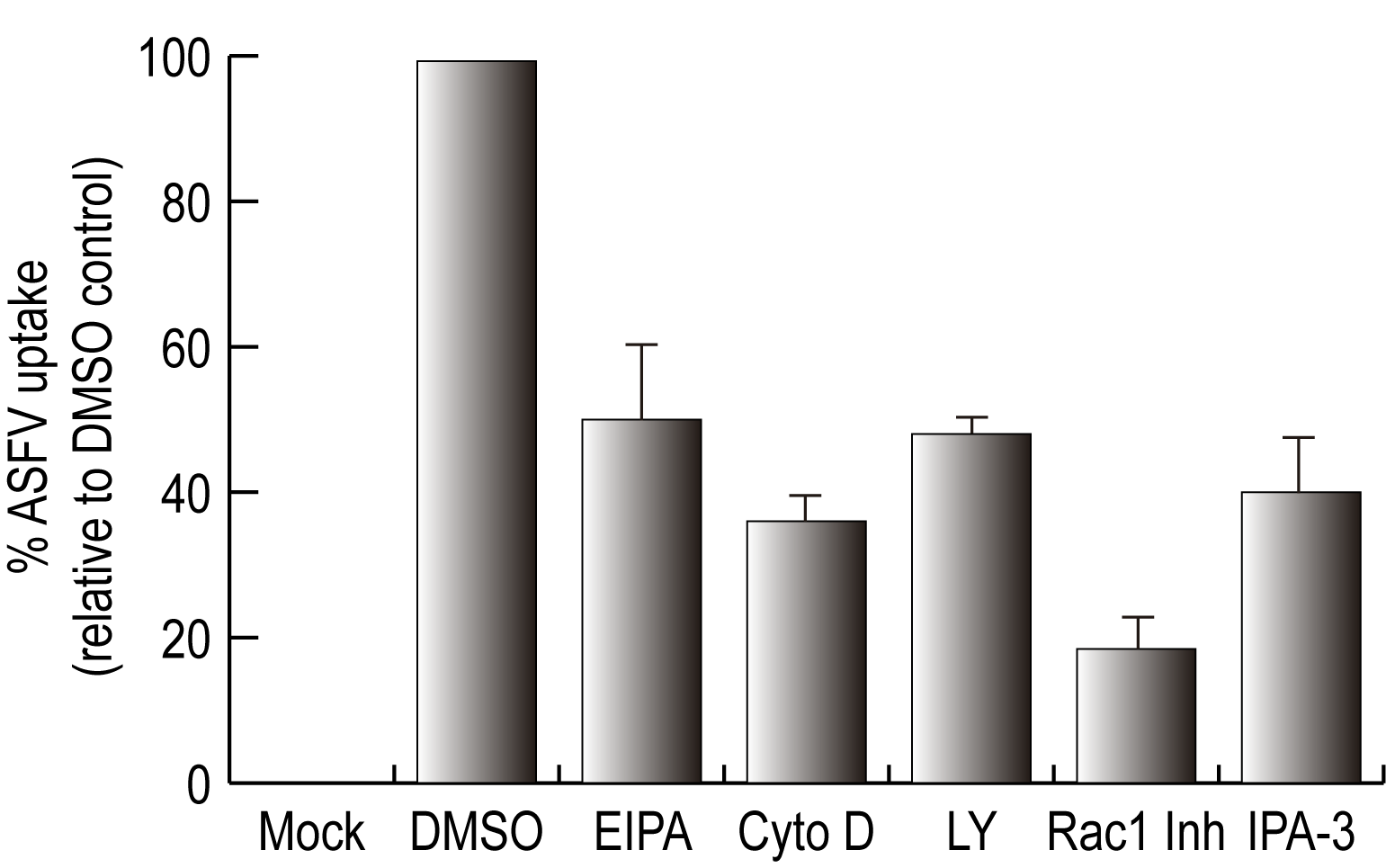

Supplement: Figure S3 — Effect of macropinocytosis inhibitors on ASFV uptake. Vero cells were pretreated with DMSO or different pharmacological inhibitors for 60 min at 37°C as follows: 60 µM EIPA, 8 µM Cyto D, 60 µM LY, 200 µM Rac1 Inh and 30 µM IPA-3. Cells were synchronously infected (MOI 10) for 60 min in the presence of the drugs, fixed and stained with Topro3 (blue), phalloidin (red) and anti-p72 (green). Images were taken by CLSM and represented as a maximum z-projection of horizontal slices (x–y plane). The LSM images were imported to Image J program and the number of virus particles inside the cells was automatically counted with a Macro algorithm in which threshold Intermodes was used to define a single virus particle in the cell. The graph shows percentage of virus inside the cells relative to DMSO control of the three independent experiments (mean ±S.D.). S.D., standard deviations; Cyto D, Cytochalasin D; LY, LY294002; Rac1 Inh, Rac1 inhibitor. (TIF) [file ppat.1002754.s003.tif]

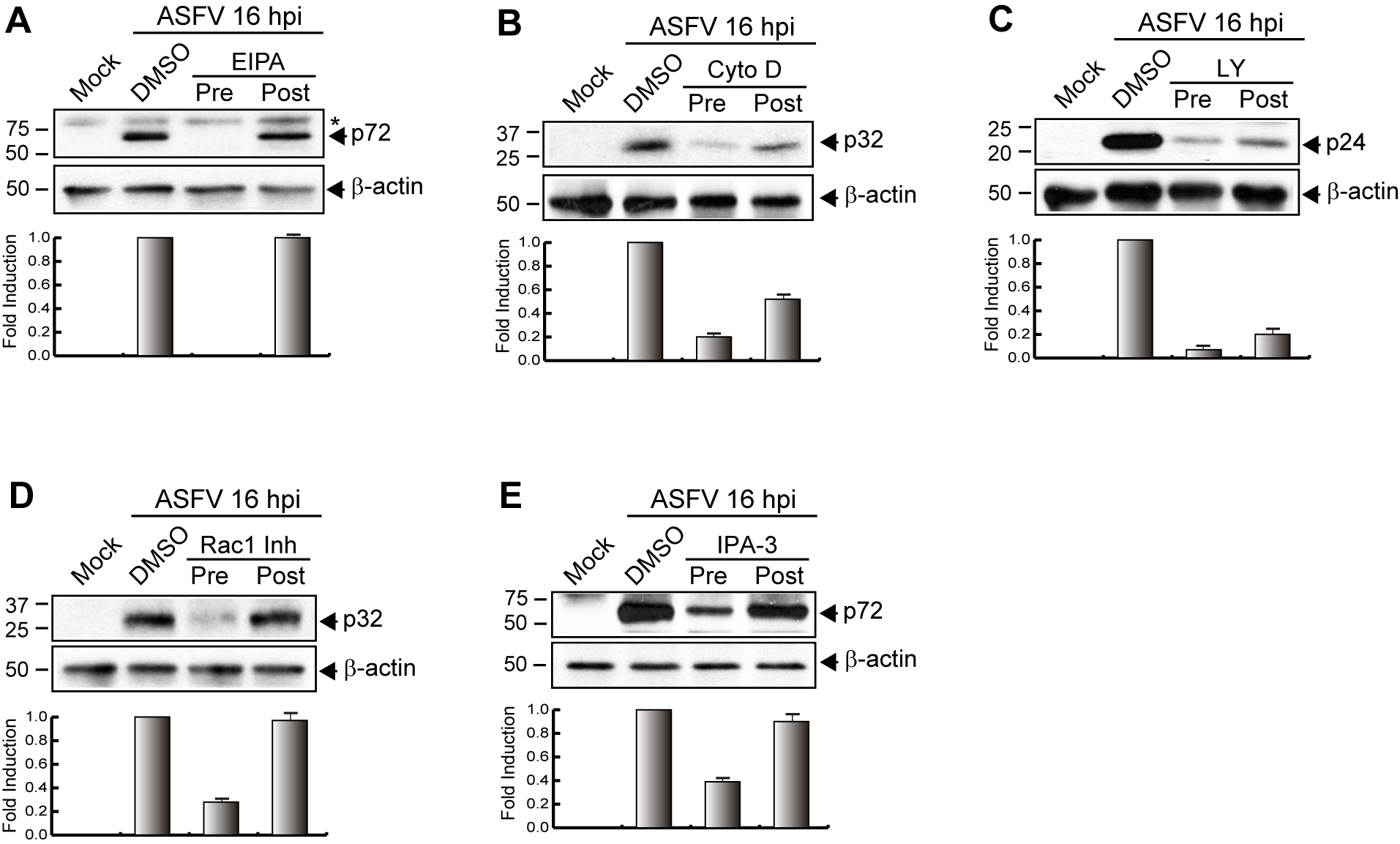

Supplement: Figure S4 — Effect of macropinocytosis inhibitors on virus entry and post entry steps. Vero cells were treated with 20 µM EIPA (A), 4 µM Cyto D (B), 20 µM LY (C), 200 µM Rac1 Inh (D) and 10 µM IPA-3 (E) for 60 min before the virus addition (Pre-treatment, Pre), or 60 min after virus addition (Post-treatment, Post), and viral infection was allowed in the presence of the drugs at 37°C, in each case. After 16 h, the cells were lysed in RIPA modified buffer and the viral proteins were analyzed by Western blot with an anti-ASFV antibody. β-actin was detected as a load control. Fold induction was determined by densitometry and represented in the graphics below (mean ±S.D.) Cyto D, Cytochalasin D; LY, LY294002; Rac1 Inh, Rac1 inhibitor. * Unspecific cellular protein detected by the antibody. (TIF) [file ppat.1002754.s004.tif]

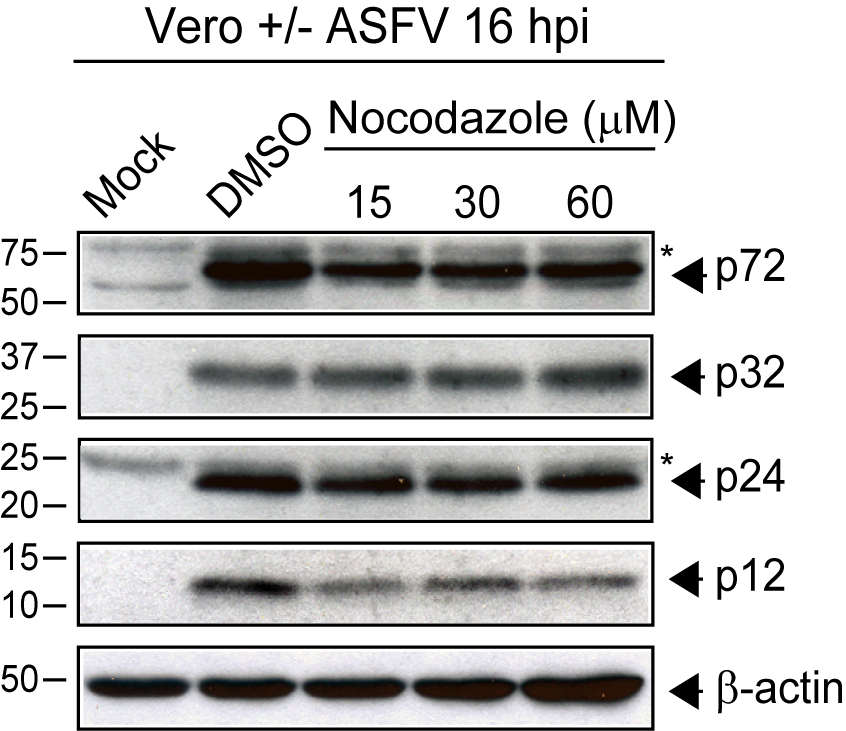

Supplement: Figure S5 — ASFV entry is not dependent on microtubule system. Vero cells were treated with nocodazole at indicated concentrations and infected with Ba71V (MOI 1) for 16 h. Viral protein synthesis was analyzed by Western blot with an anti-ASFV antibody. β-actin was detected as a load control. * Unspecific cellular protein detected by the antibody. (TIF) [file ppat.1002754.s005.tif]

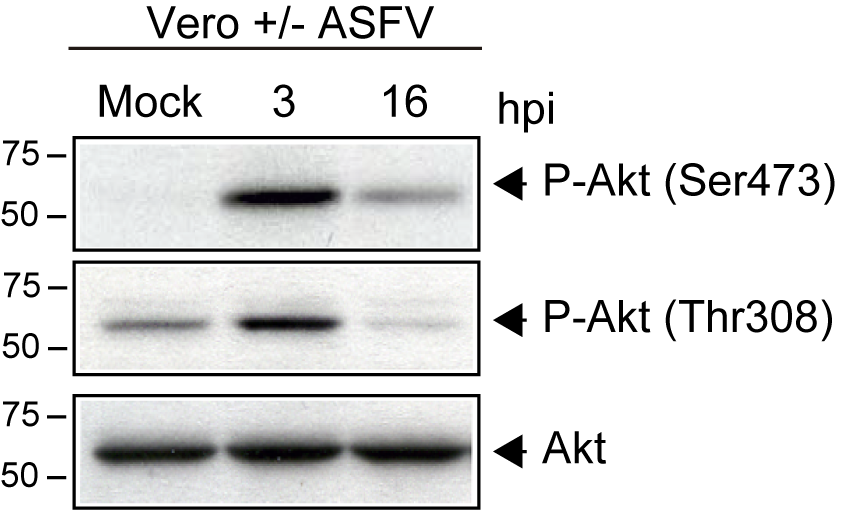

Supplement: Figure S6 — ASFV infection induces Akt phosphorylation at early time post infection. Vero cells were asynchronously infected (MOI 5) and solubilised in RIPA buffer at the indicated times post infection. Equivalent amounts of protein were analyzed by immunolotting and the phosphorylation level of Akt was analyzed by using specific antibodies against phospho-Akt Ser473 and phospho-Akt Thr308. Levels of total Akt were measured as a control. (TIF) [file ppat.1002754.s006.tif]

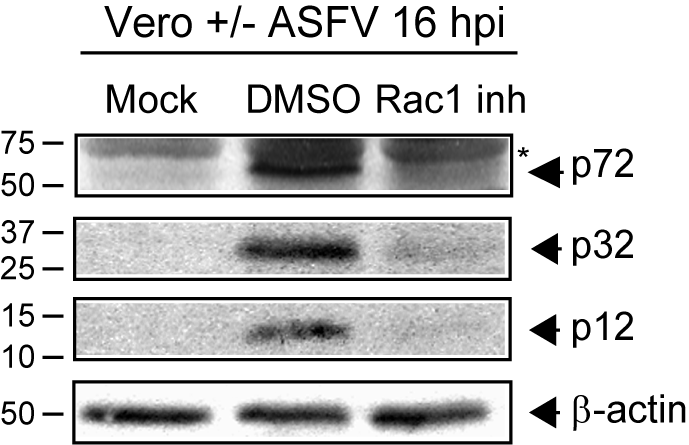

Supplement: Figure S7 — Rac1 inhibitor effect on viral proteins synthesis. Vero cells were treated with 200 µM Rac1 inhibitor and infected with Ba71V (MOI 1) for 16 h. Samples were solubilised in RIPA buffer and equivalent amounts of protein were analyzed by Western blot with an anti-ASFV antibody. β-actin was detected as a load control. * Unspecific cellular protein detected by the antibody. (TIF) [file ppat.1002754.s007.tif]

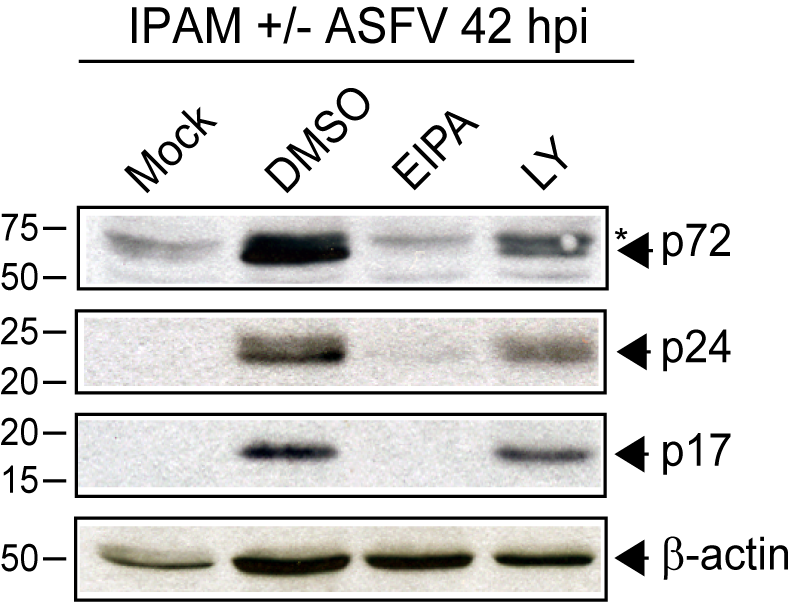

Supplement: Figure S8 — Factors involved in macropinocytosis during the infection in IPAM cells. IPAM cells were treated with EIPA and LY (20 µM both) and infected with the isolate E70 during 42 h. Viral protein synthesis was analyzed by Western blot with an anti-ASFV antibody. β-actin was detected as a load control. * Unspecific cellular protein detected by the antibody. (TIF) [file ppat.1002754.s008.tif]
